# Computational Mueller matrix microscopic pathology based on differential decomposition for medical reference mapping

**DOI:** 10.3389/fbinf.2026.1953765

**Published:** 2026-10-26

**Authors:** Yuxuan Mao, Irene Estevéz, Armando Pérez-Torres, Juan Campos, Angel Lizana

**Affiliations:** 1 Department of Physics, Optics Group, Universitat Autònoma de Barcelona, Bellaterra, Spain; 2 Cellular and Tissue Biology Department, Faculty of Medicine, Universidad Nacional Autónoma de México, Mexico, Mexico

**Keywords:** computational bioimaging, differential Mueller matrix decomposition, image stitching, multi-parametric pseudo-color mapping, polarimetric imaging

## Abstract

**Introduction:**

Label-free quantitative polarimetric imaging provides access to physically interpretable tissue properties that are not available from conventional intensity-based microscopy, offering new opportunities for computational digital pathology and tissue bioinformation analysis. By characterizing the interaction between polarized light and tissue microstructure, Mueller matrix imaging enables spatially resolved quantification of optical mechanisms including diattenuation, birefringence, and depolarization. However, translating this information toward tissue-scale pathological assessment remains challenging because high spatial resolution is inherently associated with a limited field of view, while the resulting high-dimensional polarimetric information is difficult to interpret directly and relate to conventional medical references.

**Methods:**

Here, we present a physics-informed computational framework, termed Computational Mueller Matrix Microscopic Pathology, based on differential Mueller matrix decomposition. The framework integrates differential decomposition, wide-field Mueller matrix image stitching, and mechanism-specific multi-channel pseudo-color visualization into a unified computational pipeline. By combining physically interpretable polarimetric observables with a SIFT-based stitching strategy, the framework extends high-resolution Mueller matrix imaging to large tissue areas while preserving the spatial consistency and physical interpretability of the reconstructed polarimetric information. Mechanism-specific pseudo-color mappings further transform the decomposed optical responses into spatially distinguishable representations, facilitating their visual and quantitative comparison with conventional histological references.

**Results:**

The framework was evaluated on four paired H&E-stained murine melanoma specimens under untreated (Trt^−^) and treated (Trt^+^) conditions through complementary quantitative, physical, and histological analyses. For these thin pathological sections, differential decomposition approaches and Lu–Chipman decomposition yielded comparable tissue representations, while mechanism-specific pseudo-color mappings enabled the resulting polarimetric contrasts to be visualized according to their underlying optical mechanisms. Notably, depolarization consistently emerged as a tumor-associated contrast across the polarimetric numerical distributions, spatial heterogeneity, and visual enhancement of differential pseudo-color mappings, with preliminary spatial correspondence to H&E-defined tissue regions. These observations indicate that physically interpretable polarimetric contrasts can capture spatially resolved information associated with tissue structural characteristics.

**Discussion:**

Overall, the results demonstrate the feasibility of combining differential Mueller matrix decomposition, large-field polarimetric imaging, and mechanism-specific pseudo-color visualization for tissue-scale pathological assessment. Beyond enhanced visualization, the proposed framework establishes quantitative links between histological appearance and physically interpretable optical properties, providing a foundation for objective tissue characterization and the extraction of biologically relevant spatial information. These findings suggest that Mueller matrix polarimetry, combined with high-magnification large-field imaging and differential decomposition, can provide spatially resolved polarimetric features relevant to the characterization of melanoma-associated tissue alterations and treatment-related structural changes.

## 1 Introduction

In the modern medical framework, microscopic samples, such as tissue sections and cytological smears, serve as the cornerstone of disease diagnosis and pathological research. From the characterization of inflammatory profiles to the precise staging of malignancies, microscopic pathological analysis provides the most direct evidence for clinical decision-making (Gurcan et al., 2009; Titford, 2009; Chen et al., 2011). Complementing this morphology-centered paradigm, the introduction of medical bioinformatics—benefiting from advances in specimen preservation, digitization, novel imaging technologies, and growing computational capacity for large-scale data—enables quantitative feature extraction, multi-source information fusion, and data-driven pattern recognition that offer a more comprehensive understanding of disease (Peng et al., 2012; Hassan et al., 2026).

Driven by escalating clinical demands, computational imaging methods (Isse et al., 2012; Madabhushi and Lee, 2016; Banerji and Mitra, 2022) built upon emerging optical modalities are being progressively translated into practice, advancing toward paradigms that are real-time, *in vivo*, non-invasive, multi-dimensional, and physically referenced (Fuchs and Buhmann, 2011). For microscopic pathology, non-invasiveness and physical interpretability are the most pressing yet frequently conflicting requirements. Fluorescent labeling and chemical staining do more than enhance visual contrast—they alter the specimen’s native chemical and physical state; laser-scanning modalities such as confocal microscopy further impose measurable phototoxicity (Mait et al., 2018). Meanwhile, the added information from multi-dimensional imaging cannot be trusted in isolation, requiring corroboration against clinical references such as expert diagnosis or supervised deep learning. Together, these constraints reveal a critical bottleneck: downstream bioinformatic sophistication (Wu et al., 2012; Gullapalli et al., 2012) is ultimately bounded by the physical fidelity of the upstream imaging modality. Realizing precision pathology’s full potential therefore requires an imaging modality that is non-destructive, compatible with unstained specimens, and capable of resolving quantitative, physically interpretable microstructural information.

Mueller Matrix (MM) microscopy (Arteaga et al., 2014; He et al., 2019; Ma et al., 2022) has emerged as a powerful computational imaging tool, offering a comprehensive description of the interaction between polarized light and specimens. Building upon the Stokes-Mueller formalism, Mueller matrix polarimetry fully encodes the polarimetric properties of the sample, allowing for the extraction of physical parameters such as dichroism, birefringence, and depolarization (Goldstein 2017; Gil and Ossikovski, 2022)—parameters reflecting the shape, density, and spatial distribution of the observed objects in a label-free and quantitatively reproducible manner. The broader field of polarization-based optical microscopy (Wolman, 1975; He et al., 2021) has a well-established history in biological imaging, rooted in the fundamental observation that biological tissues are inherently anisotropic media. Structural proteins such as collagen (Golaraei et al., 2019), myelin (Blanke et al., 2023), and actin filaments (Curcio et al., 2020) exhibit intrinsic optical anisotropy arising from their ordered molecular architecture, rendering them naturally amenable to polarimetric contrast mechanisms. Over the past 2 decades, polarimetric microscopic imaging has progressively demonstrated its clinical relevance across a broad spectrum of biological and pathological investigations. In oncological histology, Mueller matrix imaging methods have been applied to image collagen birefringence in normal human skin, melanoma, basal cell carcinoma, and squamous cell carcinoma (Ceolato et al., 2015; Vahidnia et al., 2021; Le and Bolden, 2025), with retardance images successfully highlighting clusters of collagen fibers in tumorous skin. Furthermore, analyzing collagen fiber orientation as a function of depolarization proves useful for categorizing distinct types of skin cancer. In gynecological pathology, quantitative Mueller matrix polarimetric imaging captures essential morphological information regarding cervical tissue sections (Shukla and Pradhan, 2009)—in particular, the disruption of anisotropically organized collagen fibers that occurs during malignant transformation (Chue-Sang et al., 2017; Ramella-Roman and Ajmal, 2024), enabling non-invasive distinction between healthy and pathological cervical tissue.

To extract the rich physical information encoded in the Mueller matrix, conventional interpretation methods aim to recover meaningful parameters by imposing physically motivated constraints. Representative techniques, such as the Lu–Chipman decomposition (Lu and Chipman, 1996), symmetric decomposition (Ossikovski, 2009), and arrow decomposition (Gil, 2013), assume specific interaction sequences to ensure the physical feasibility of the matrix product and decompose the Mueller matrix accordingly. These product-based methods inherently assume the specimen’s polarization response to arise from a discrete sequence of physically distinct interactions, and they have proven effective for bulk tissue samples. It should be noted that product-based methods, remain robust and useful tools for extracting phenomenological polarization features from thin tissue slides. Nevertheless, because anisotropic and scattering events in biological tissue are inherently distributed and intertwined, the continuous-medium formulation of differential Mueller matrix decompositon (Ortega-Quijano and Arce-Diego, 2011; Arteaga and Kahr, 2013; Ossikovski and Arteaga, 2014) offers a more physically matched alternative, since microscopic pathological samples are typically prepared as such thin sections. The differential Mueller matrix decomposition yields a mathematically unique log-matrix solution that avoids the non-commutative factor-order dependency of product decompositions. This unique mapping allows the full polarimetric capacity of thin specimens to be comprehensively mapped into a rich array of independent physical channels, including linear and circular dichroism, linear and circular birefringence, and isotropic absorption and statistical form depolarization. Although introduced more recently than conventional decompositions, this emerging approach is gaining rapid traction in the biomedical field, where ongoing clinical and pathological studies continue to validate its rich diagnostic value (Kumar et al., 2024).

While differential decomposition provides a more physically faithful inversion of polarimetric parameters at the microscopic scale, translating these parameters into a clinically actionable diagnostic tool requires addressing two further challenges that extend beyond single-field-of-view (FOV) parameter extraction. First, pathological diagnosis is inherently multi-scale (Brenes et al., 2024): pathological survey overall tissue architecture at low magnification to localize regions of interest—such as tumor margins or invasion fronts—before examining them at high magnification for cellular- and fiber-level detail, yet the magnification required to resolve microscopic polarimetric features inherently restricts the FOV. Medical image stitching (Januszewski et al., 2024), which reconstructs a panoramic FOV by seamlessly registering and merging overlapping sub-images, addresses this by enabling whole-slide imaging (WSI) (Farahani et al., 2015) that preserves microscopic resolution while supporting this multi-scale diagnostic workflow (Zhao et al., 2010). Second, although polarimetric parameters provide quantitatively measurable and physically interpretable information, their direct visualization is less familiar in a histopathological context. Pseudo-color mapping (Semary, 2018; Canabal-Carbia et al., 2024) offers a physics-informed visual enhancement by integrating complementary channels into intuitive color representations. Combined with medical reference images such as H&E histology, this representation can facilitate the interpretation of spatial polarimetric variations in relation to tissue morphology and pathological features, based on the physical meaning of the underlying channels and their previously reported biological associations. Such an approach may improve the accessibility of polarimetric information for histopathological interpretation, although its clinical relevance ultimately requires validation across larger cohorts and with expert pathological assessment.

In summary, this paper presents a comprehensive physics-informed computational framework termed Computational Mueller Matrix Microscopic Pathology. To the best of our knowledge, this represents the first integration of Mueller matrix differential decomposition, wide-field image stitching, and multi-channel pseudo-color visualization into a unified framework for quantitative histological slide characterization. Building upon a Mueller matrix microscopy system, a rigorous digital processing strategy is implemented in which physically interpretable polarimetric observables are extracted from high-resolution, wide-FOV acquisitions of histological specimens of murine melanoma and subsequently translated into intuitive pseudo-color representations through mechanism-specific visualization schemes. Beyond enhancing visual interpretation, the proposed framework provides a means of relating spatial variations in polarimetric parameters to tissue morphology through the underlying optical mechanisms of dichroism, birefringence, and depolarization. These physically interpretable descriptors enable the investigation of tissue heterogeneity within the tumor microenvironment (Sprenger et al., 2021; Lad et al., 2022) and facilitate quantitative comparisons between treatment conditions. The results obtained in this work demonstrate the feasibility of integrating Mueller matrix imaging stitching and pseudo-color visualization for computationally assisted histological characterization after quantitatively assess.

Specially, the histopathological sections analyzed in this work were obtained from a well-established preclinical mouse melanoma model induced by a validated melanoma cell line, which reproduces several key features of human melanoma. Administration of the GK-1 peptide and AGRO aptamer as potential antitumor agents can induce treatment-associated alterations in the tumor microenvironment, including changes in cellular morphology, apoptosis/necrosis, and vascularity that can be observed by H&E staining. Rather than serving a diagnostic purpose, this model provides a controlled setting for evaluating label-free imaging of treatment-related histopathological changes. In this context, polarimetric false-color imaging offers complementary, physically interpretable contrast without additional labeling, with potential utility for characterizing tumor-microenvironment alterations in preclinical cancer studies, including the evaluation of emerging antitumor therapies.

## 2 Methodology

### 2.1 Mueller matrix imaging principle

The interaction of polarized light with biological specimens is mathematically captured by the Stokes-Mueller formalism. In a typical polarimetric setup, this optical process is governed by two functional blocks—the Polarization State Generator (PSG) and the Polarization State Analyzer (PSA)—and the detected intensity matrix I is expressed as:

I=AMW (1)

where $W\in R^{4\times n_{g}}$ represents the $n_{g}$ pairs of generated Stokes vectors of the PSG, $A\in R^{n_{a}\times 4}$ denotes the $n_{a}$ pairs of analyzed polarized states using the PSA, and $M\in R^{4\times 4}$ is the sample’s Mueller matrix. The measurement fidelity and noise robustness of the polarimeter are strictly dictated by the configurations of A and W . Mathematically, the amplification of experimental noise during inversion is bounded by the condition number (CN) (Twietmeyer and Chipman, 2008) of the generator matrix and analyzer matrix. To experimentally realize the sets of polarization states encoded in W and A , one of the most common implementations is the division-of-time scheme (Farlow et al., 2002), in which the PSG and PSA are sequentially modulated to generate the $n_{g}\times n_{a}$ combinations of incident and analyzing states required (typically via rotating retarders or liquid-crystal variable retarders), with the corresponding intensities recorded frame-by-frame and used to reconstruct the Mueller matrix M . To achieve fast processing for large-scale pixel arrays, Equation 1 is routinely vectorized utilizing the Kronecker product (Zallat et al., 2006; Chipman et al., 2018). By applying the identity $\text{vec}\left(ABC\right)=\left(C^{T}⊗A\right)\text{vec}\left(B\right)$ , the forward model is linearized into $\text{vec}\left(I\right)=\left(W^{T}⊗A\right)\text{vec}\left(M\right)$ , enabling an efficient, single-step pixel-wise reconstruction:

$$\text{vec}\left(M\right)={\left(W^{T}⊗A\right)}^{+}\text{vec}\left(I\right)$$ (2)

where ${\left(\cdot \right)}^{+}$ denotes the Moore-Penrose pseudo-inverse.

### 2.2 Differential Mueller matrix decomposition

Compared to mature and widely used Mueller matrix decomposition techniques (such as the Lu–Chipman decomposition), the Mueller matrix differential decomposition represents a more recent tool (Ortega-Quijano and Arce-Diego, 2011). The primary motivation for adopting this novel approach is to investigate whether, in experimental scenarios that closely align with its underlying physical assumptions—namely, the continuous and simultaneous occurrence of multiple polarization effects—it can achieve a parameter characterization capability comparable to that of conventional techniques. Technically, the differential decomposition maps the macroscopically measured Mueller matrix M using Equation 2 to a differential matrix L , which represents the polarimetric properties per unit path length via the matrix logarithm:

$$L=\ln\left(M\right)$$ (3)

Utilizing the Minkowski metric tensor G=diag(1,−1,−1,−1) , the differential matrix L can be uniquely decoupled into an antisymmetric component $L_{m}$ and a symmetric component $L_{u}$ :

$$L_{m}=\frac{1}{2}\left(L-GL^{T}G\right),\;L_{u}=\frac{1}{2}\left(L+GL^{T}G\right)$$ (4)

The non-depolarizing component $L_{m}$ encapsulates the six elementary optical properties governed by deterministic anisotropic structures, namely, dichroism (D) and birefringence (B) :

$$L_{m}=\left[\begin{array}{cccc} 0 & -LD & -LD^{\prime } & CD\\ -LD & 0 & CB & LB^{\prime }\\ -LD^{\prime } & -CB & 0 & -LB\\ CD & -LB^{\prime } & LB & 0 \end{array}\right]$$ (5)

where LD and $LD^{\prime }$ represent linear dichroism (selective absorption) along the 0°-90° and ±45° axes, respectively, while CD represents circular dichroism. Concurrently, LB and $LB^{\prime }$ denote linear birefringence, and CB denotes circular birefringence. Conversely, following the statistical interpretation of polarimetric fluctuations (Ossikovski and Arteaga, 2014), the depolarizing component $L_{u}$ arises as a direct consequence of the spatial or temporal variations of these six elementary properties. The algebraic structure of $L_{u}$ is expressed as:

$$L_{u}=\left[\begin{array}{cccc} A & \Delta p_{1} & \Delta p_{2} & \Delta p_{3}\\ -\Delta p_{1} & A-LDP & \Delta p_{4} & \Delta p_{5}\\ -\Delta p_{2} & \Delta p_{4} & A-LDP^{\prime } & \Delta p_{6}\\ -\Delta p_{3} & \Delta p_{5} & \Delta p_{6} & A-CDP \end{array}\right]$$ (6)

where A signifies the isotropic absorption, while LDP , $LDP^{\prime }$ , and CDP correspond to the linear and circular depolarization rates, respectively. The anomalous cross-projections $\Delta p_{i}$ serve as an index of structural inhomogeneity or time-reversal symmetry breaking (e.g., local depolarization gradients or fluorescence contributions); in a perfectly homogeneous medium, these terms vanish $\left(\Delta p_{i}\approx 0\right)$ . However, in the presence of non-reciprocal depolarizing mechanisms—such as fluorescence or inelastic scattering—a measurable, non-zero $\Delta p_{i}$ can emerge even for an otherwise homogeneous sample. Under such circumstances, directly extracting the six non-depolarizing parameters from the unmodified $L_{m}$ may yield results that violate fundamental physical constraints. To account for this effect, $L_{u}$ obtained using Equations 3, 4, 6 is further partitioned into its diagonal and off-diagonal components:

$$L_{u}=L_{u}^{d}+L_{u}^{nd}$$ (7)

where $L_{u}^{d}=diag\left(A,\;A-LDP,\;A-LDP^{\prime },\;A-CDP\right)$ represents the genuine anisotropic depolarization, while $L_{u}^{nd}$ collects the off-diagonal terms $\Delta p_{i}$ . Physically, $L_{u}^{nd}$ represents non-depolarizing optical information that has been transferred out of $L_{m}$ as a consequence of the time-irreversible mechanism, manifesting instead as anomalous depolarization terms. To recover this lost contribution, a corrected non-depolarizing matrix $L_{m}^{\prime }$ is obtained via the anticommutator of $L_{u}^{nd}$ and $L_{m}$ calculated from Equation 5:

$$L_{m}^{\prime }=L_{m}+\left\{L_{u}^{nd},L_{m}\right\}=L_{m}+L_{u}^{nd}L_{m}+L_{m}L_{u}^{nd}$$ (8)

The six elementary optical parameters— LD , $LD^{\prime }$ , CD , LB , $LB^{\prime }$ , and CB —are subsequently extracted from $L_{m}^{\prime }$ rather than the uncorrected $L_{m}$ , ensuring that the recovered dichroic and birefringent properties remain physically self-consistent and Kramers–Kronig compliant, even in the presence of non-negligible $\Delta p_{i}$ contributions. To provide a comprehensive and structured overview of this computational framework, the extracted differential polarimetric parameters according to Equations 5, 6 and their corresponding physical interpretations are summarized in Table 1.

**TABLE 1:** Summary of derived differential Mueller matrix parameters.

Category	Parameter	Symbol	Physical/Structural significance
Dichroism	Linear dichroism	$LD,LD^{\prime }$	Selective absorption of linear states along 0°/90° and ±45° axes
	Circular dichroism	CD	Selective absorption of left- and right-circularly polarized states
Birefringence	Linear birefringence	$LB,LB^{\prime }$	Phase retardance along 0°/90° and ±45° axes
	Circular birefringence	CB	Phase retardance between left- and right-circularly polarized states
Depolarization	Isotropic absorption	A	Global attenuation of light intensity during propagation
	Linear depolarization	$LDP,LDP^{\prime }$	Depolarization rate for 0°/90° and ±45° linearly polarized light
	Circular depolarization	CDP	Depolarization rate for circularly polarized light

### 2.3 Downstream digital processing: stitching and pseudo-coloring

In this work we use polarization microscopy for the study of samples, with particular emphasis on biological samples, although the methods are applicable to any sample that can be measured by microscopy and is sensitive to polarization. In this context, once the system is capable of measuring the microscopic Mueller matrix, we combine and apply two methods that allow us to go a step further in the image processing of the images obtained. First, we develop a polarimetric image stitching approach that extends the field of view while preserving the spatial resolution provided by high-magnification objectives, thereby addressing the inherent trade-off between spatial resolution and field of view in conventional microscopy. Second, we introduce a multi-channel pseudo-color visualization strategy that jointly represents complementary polarimetric observables. By mapping physically meaningful polarimetric channels to different color components, the proposed representation enhances the visualization of tissue structures and facilitates the interpretation of spatial variations in the underlying polarimetric properties.

#### 2.3.1 Wide FOV realization: intensity-guided stitching

In this subsection, we present a reconstruction framework that fuses multi-tile polarimetric FOVs into a seamless and panoramic Mueller matrix imaging. The central idea of this framework is a two-stage strategy: geometric registration is performed entirely in the intensity domain, while the resulting spatial transformations are subsequently applied to other Mueller matrix elements. This design choice is motivated by the fact that registration algorithms (e.g., feature detection and matching) are most reliable when operating on intensity-like images, thereby avoiding the complex, spatially varying polarimetric artifacts that could otherwise confound feature detection. To implement this strategy, for each tile at row r and column c , we extract the total intensity map directly from the $M_{00}$ element of the reconstructed Mueller matrix, $I_{r,c}=M_{00}\left(r,c\right)$ , and apply standard histogram equalization (Gonzalez, 2009; Mustafa and Kader, 2018) to obtain a contrast-enhanced reference map $I_{r,c}^{*}$ suitable for keypoint extraction.

In addition, pairwise registration between adjacent FOVs is then performed using SIFT (Lowe, 2004) feature matching combined with RANSAC (Fischler and Bolles, 1981) (minimum match criterion $n_{\text{match}}\geq 3$ ), yielding a local 3×3 affine transformation $T_{\Delta ,r,c}$ that corrects for small misalignments between neighboring tiles introduced by motorized stage. Under this scenario, the transformation matrix $T_{\Delta ,r,c}$ takes the form:

$$T_{\Delta ,r,c}=\left[\begin{array}{ccc} a_{1} & a_{2} & 0\\ a_{3} & a_{4} & 0\\ t_{x} & t_{y} & 1 \end{array}\right]$$ (9)

Each local correction $T_{\Delta ,r,c}$ is composed with the nominal step-translation matrices $T_{n,H}$ and $T_{n,V}$ . These nominal matrices are derived from the predefined raster scanning protocol of the motorized stage, where $W_{0}$ and $H_{0}$ denote the tile dimensions and α represents the controlled overlap ratio. By incorporating these known physical parameters, the model effectively constrains the registration process to the scanning grid:

$$T_{n,H}=\left[\begin{array}{ccc} 1 & 0 & 0\\ 0 & 1 & 0\\ W_{0}\cdot \alpha & 0 & 1 \end{array}\right],\;T_{n,V}=\left[\begin{array}{ccc} 1 & 0 & 0\\ 0 & 1 & 0\\ 0 & H_{0}\cdot \alpha & 1 \end{array}\right]$$ (10)

and the global transformation for tile (r,c) is obtained recursively as:

$$T_{r,c}=T_{\Delta ,r,c}\cdot T_{n,H}^{\;c}\cdot T_{n,V}^{\;r}\cdot T_{0,0}$$ (11)

where $T_{0,0}$ denotes the placement of the reference tile at the origin of the scanning grid. This recursive formulation ensures that each tile’s position is consistently anchored to the global grid rather than drifting based on local pairwise estimates alone.

Local pixel coordinates $X_{L}=\left[x_{L},y_{L},1\right]$ are mapped to global panoramic coordinates $X_{G}=\left[x^{\prime },y^{\prime },1\right]$ via

$$X_{G}=X_{L}\cdot T_{r,c}$$ (12)

and the bounding dimensions of the panoramic canvas, ${\text{Canvas}}_{X}$ and ${\text{Canvas}}_{Y}$ , are determined by tracking the extremal corner projections across all tiles, ensuring no spatial truncation occurs during rendering.

Finally, as noted above, $T_{r,c}$ —though calculated based on Equations 9–11 derived solely from the intensity reference maps $I_{r,c}^{*}$ —is applied identically to all sixteen Mueller matrix elements of each tile using Equation 12. This guarantees a complete, spatially continuous polarimetric mosaic spanning the entire tissue section. While the resulting panoramic mosaic ensures that no spatially relevant tissue structure is lost to a limited field of view, the sixteen raw Mueller matrix elements—and the differential parameters derived from them—remain physically abstract quantities with no direct visual counterpart in conventional histology.

#### 2.3.2 Physics-informed translation: pseudo-color mapping

Raw Mueller matrix elements and derived parameters provide a visualization of quantitative physical information, with ranges that depend on the specific channel under study. As in other applications, the use of pseudo-color techniques allows this data to be translated into a physical interpretation in which different colors weight the predominance of a specific physical characteristic within an image. The proposed pseudo-color representation is designed to combine physically distinct and complementary polarimetric observables, providing channel-specific contrast associated with different polarization responses of the tissue. Importantly, these polarization responses are not independent of tissue organization, but can be influenced by structural properties such as tissue density, characteristic feature size, and the orientation and organization of anisotropic structures. Consequently, the resulting pseudo-color patterns can provide a physically informed representation of tissue heterogeneity that complements conventional medical reference images, such as H&E histology. In regions identified by H&E as pathological or morphologically distinctive, the corresponding polarimetric features may provide additional insight into the optical and structural mechanisms underlying the observed tissue changes. Moreover, pseudo-color representations may reveal polarimetric features extending beyond those explicitly identified in conventional histological annotations, thereby providing additional information for subsequent pathological investigation.

In particular, we have applied a polarimetric-parametric pseudo-color mapping that prioritizes the simultaneous visualization of orthogonal physical traits within a single co-registered view. Rather than encoding spectral information, this scheme assigns three derived differential parameters—each sensitive to a distinct polarization-altering mechanism—to the R, G, and B channels. We have selected two complementary strategies for pseudo-coloring, whose specific enhacement depend on the characteristics of the sample under study. The first approach, inter-mechanism mapping, assigns one representative parameter from each one of the three main polarization-altering features of a sample—dichroism, birefringence, and depolarization—providing a comprehensive, multi-mechanism view of the sample’s microstructural composition. The second approach, intra-mechanism mapping, assigns the linear (horizontal/vertical and ±45° ) and circular components of a single mechanism to the RGB channels; for example, the three retardance components of birefringence (or analogously, of dichroism) can be jointly visualized to highlight orientation- and chirality-dependent features, such as the directionality of collagen fibers, that would otherwise be averaged out in a single scalar parameter. In both cases, the chosen parameter triplet can be regarded as defining a three-dimensional physical feature space $q=\left(q_{1},q_{2},q_{3}\right)$ that captures the dominant polarization-altering behavior of the sample. To render this abstract space visually, a color-encoding map Φ rescales each axis independently to [0, 1] via min-max normalization N(⋅) and identifies it with one channel of the RGB color cube:

$$\Phi :q=\left(q_{1},q_{2},q_{3}\right)⟼C_{\text{polarimetric}}=\left(N\left(q_{1}\right),\;N\left(q_{2}\right),\;N\left(q_{3}\right)\right)\in {\left[0,1\right]}^{3}$$ (13)

This per-channel normalization effectively performs a change of basis from heterogeneous physical units to a common perceptual scale, so that each axis of the physical feature space contributes comparably to the final hue, regardless of its native dynamic range.

### 2.4 Quantitative evaluation metrics

To systematically evaluate both the algorithmic fidelity of Mueller matrix image stitching and the visual enhancement and preliminary histopathological relevance of the proposed polarimetric mapping, we implemented a dual-stage quantitative evaluation framework. On the one hand, the proposed Mueller matrix image stitching aims to achieve a large FOV while preserving the high spatial resolution required for microscopic polarimetric imaging. Accordingly, spatial and visual continuity across tile boundaries, together with the fidelity of the reconstructed physical polarimetric parameters, constitute the key requirements for evaluating the stitching performance. On the other hand, the primary objective of the proposed polarimetric mapping is to establish a digital virtual-staining representation based on physically interpretable polarimetric information. Since the differential decomposition provides multiple physically distinct and complementary observables, different pseudo-color mappings can emphasize different aspects of tissue organization. The evaluation therefore focuses on the resulting visual enhancement and on how different mappings exhibit visual feature enhancement, together with a preliminary assessment of their correspondence with H&E-derived tissue features.

#### 2.4.1 Performance evaluation of wide-field image stitching

To rigorously assess the quality and seamlessness of our stitched wide-field images, we established a progressive, dual-level evaluation pipeline comprising two complementary metrics:

1. Photometric Discrepancy (Local Seam Continuity): For each pair of horizontally or vertically adjacent tiles, the stitching seam is determined from the SIFT-based spatial registration. The overlapping region shared by the two source tiles is consequently divided by the estimated seam into two corresponding sub-regions, denoted as $\Omega_{A}$ and $\Omega_{B}$ , which originate from the two adjacent acquisitions, respectively. Photometric consistency is evaluated directly on the raw Mueller matrix elements by quantifying the differences in mean and standard deviation across these two seam-adjacent regions (Brown and Lowe, 2007):

$${PC}^{\left(i,j\right)}=\frac{\left|\mu_{A}^{\left(i,j\right)}-\mu_{B}^{\left(i,j\right)}\right|+\left|\sigma_{A}^{\left(i,j\right)}-\sigma_{B}^{\left(i,j\right)}\right|}{\mu_{A}^{\left(i,j\right)}+\mu_{B}^{\left(i,j\right)}},$$ (14)

where $\mu_{A}^{\left(i,j\right)}$ and $\sigma_{A}^{\left(i,j\right)}$ denote the mean and standard deviation of the raw $M_{ij}$ element within region $\Omega_{A}$ , respectively, and $\mu_{B}^{\left(i,j\right)}$ and $\sigma_{B}^{\left(i,j\right)}$ are the corresponding statistics within $\Omega_{B}$ . The superscript (i,j) explicitly indicates that the metric is independently evaluated for each Mueller matrix element. Lower ${PC}^{\left(i,j\right)}$ values indicate greater photometric consistency of the corresponding $M_{ij}$ channel between the two source tiles and, consequently, reduced discontinuity across the stitching seam. This metric evaluates channel-wise local continuity at tile boundaries directly on the raw Mueller matrix elements, without additional normalization or cross-scale comparison.

2. Cross-Scale MAE (Global Reconstruction Fidelity): To evaluate whether the high-magnification stitched reconstruction preserves the quantitative Mueller matrix information observed in the same region without involving stitching, we compare registered corresponding sub-regions acquired using the two imaging configurations. Specifically, the high-magnification stitched image is spatially registered to a corresponding single-FOV acquisition obtained at lower magnification, and the comparison is restricted to their common registered region. After normalization, the mean absolute error (MAE) is calculated for each Mueller matrix element $M_{ij}$ :

$$L_{\text{CS}-\text{MAE}}^{\left(i,j\right)}=\frac{1}{N}\overset{N}{\underset{p=1}{\sum }}\left|M_{\text{stitch}}^{\left(i,j\right)}\left(p\right)-M_{\text{single}}^{\left(i,j\right)}\left(p\right)\right|,$$ (15)

where $M_{\text{stitch}}^{\left(i,j\right)}\left(p\right)$ and $M_{\text{single}}^{\left(i,j\right)}\left(p\right)$ denote the normalized values of the $M_{ij}$ element at pixel p in the registered high-magnification stitched image and the corresponding lower-magnification single-FOV image, respectively, and N is the number of pixels within the common registered region. Lower values indicate better cross-scale consistency between the stitched reconstruction and the independently acquired lower-magnification reference.

#### 2.4.2 Visual effect evaluation of pseudo-color mapping

To quantitatively assess the visual enhancement provided by different pseudo-color mappings, three reference-free image-quality descriptors were calculated based on the grayscale representation of each pseudo-color image. Let I(x,y) denote the pixel intensity of the converted grayscale image $I_{\text{gray}}$ at location (x,y) .

1. GLCM contrast: The Gray-Level Co-occurrence Matrix (GLCM) (Haralick et al., 1973) was used to quantify local intensity variations and the resulting contrast of microstructural patterns. The GLCM contrast was calculated as

$$C_{\text{GLCM}}=\overset{N_{g}-1}{\underset{i=0}{\sum }}\overset{N_{g}-1}{\underset{j=0}{\sum }}{\left(i-j\right)}^{2}P\left(i,j\right),$$ (16)

where P(i,j) denotes the normalized GLCM probability of observing gray levels i and j in neighboring pixels across prescribed spatial offsets, and $N_{g}$ is the number of quantized gray levels. A higher value indicates stronger local intensity variation and greater structural contrast.

2. Shannon entropy: Shannon entropy (Gray, 2011) was used to quantify the distributional richness of intensity values:

$$H=-\overset{N_{g}-1}{\underset{i=0}{\sum }}p_{i}\log_{2}p_{i},$$ (17)

where $p_{i}$ denotes the probability of occurrence of the i th gray level in $I_{\text{gray}}$ . A higher entropy reflects a broader intensity distribution and greater information content.

3. Average gradient (AG): The average gradient (Li et al., 2017) was used to quantify local spatial intensity variations and edge sharpness. It was calculated as

$$AG=\frac{1}{MN}\overset{M}{\underset{x=1}{\sum }}\overset{N}{\underset{y=1}{\sum }}\sqrt{\frac{G_{x}{\left(x,y\right)}^{2}+G_{y}{\left(x,y\right)}^{2}}{2}},$$ (18)

where $G_{x}\left(x,y\right)$ and $G_{y}\left(x,y\right)$ represent the horizontal and vertical directional gradients of $I_{\text{gray}}$ at pixel (x,y) , respectively, and M×N denotes the image resolution. A higher AG indicates sharper structural boundaries and clearer detail definition.

Together, GLCM contrast, Shannon entropy, and average gradient provide complementary measures of local structural contrast, intensity-distribution richness, and spatial boundary sharpness, respectively.

#### 2.4.3 Cross-modality comparison with H&E histology staining

As a step toward investigating the histopathological relevance of the proposed pseudo-color polarimetric representation, an exploratory cross-modality comparison was performed against H&E histology staining, aiming to examine whether tissue regions highlighted by the polarimetric representation exhibit a measurable spatial correspondence with histologically distinctive regions.

For each H&E image, a feature mask (A) was obtained using a stain-based segmentation procedure. The image was first luminosity-standardized to reduce background and illumination variability, followed by Macenko stain unmixing (Macenko et al., 2009) to estimate slide-specific stain vectors and obtain the corresponding Hematoxylin concentration map $\left(H_{\text{OD}}\right)$ . Because the Hematoxylin channel emphasizes nuclei and hyperchromatic cellular regions, $H_{\text{OD}}$ was normalized and segmented using Otsu’s thresholding (Otsu, 1979). Morphological opening with an elliptical structuring element was subsequently applied to suppress small isolated regions, yielding the final H&E-derived feature mask A .

A corresponding feature mask (B) was independently extracted from the pseudo-color polarimetric image using SLIC superpixel segmentation (Achanta et al., 2012; Irving, 2016). By grouping spatially neighboring pixels with similar color characteristics into coherent regions, SLIC provides a region-based representation of the structures highlighted by the pseudo-color mapping while reducing sensitivity to local pixel-level fluctuations. The resulting polarimetric feature mask B was then compared with the H&E-derived feature mask A to provide a preliminary quantitative measure of their spatial correspondence.

Specifically, the Dice Similarity Coefficient (DSC) (Dice, 1945) was calculated as

$$DSC\left(A,B\right)=\frac{2|A\cap B|}{\left|A|+|B\right|}.$$ (19)

A higher DSC indicates greater overlap between the tissue regions identified by the two modality-specific feature extraction procedures. Importantly, this comparison is intended as an exploratory cross-modality assessment rather than a clinical or diagnostic validation. The DSC therefore provides an indication of spatial correspondence between polarimetric visual features and H&E-defined tissue regions.

## 3 Materials and instrumentation

### 3.1 Material: melanoma pathology

Tissue samples were obtained from a melanoma murine model approved by the Institutional Committee for the Care of Laboratory Animals (CICUAL, UNAM; approval no. 029-CIC-2020) and the Research and Bioethics Committees of the Faculty of Medicine, UNAM (approval no. FM/DI/086/2020), in accordance with Mexican (NOM-062-ZOO-1999) and ARRIVE guidelines. B16-F10 melanoma cells were subcutaneously implanted into the flank of 18 C57BL/6 male mice (6–8 weeks old), divided into a control group (saline) and a treatment group (AGRO/GK-1 combination). After tumor development (defined as day 0), mice were monitored for 3 weeks before euthanasia. Primary tumors were excised, fixed in 10% buffered formalin, paraffin-embedded, and sectioned at 4 µm for histopathological and polarimetric analysis (H&E staining). A total of 51 histological sections were obtained from 18 mice (25 sections from the control group, 26 from the treatment group) (Montes-Gonzalez et al., 2026).

The treated melanoma samples were obtained from B16-F10 tumor-bearing mice treated with a combination of AGRO (AS1411 aptamer) and GK-1 peptide. AGRO was delivered by intratumoral injection (20 µmol in 50 µL saline, three times per week), whereas GK-1 was administered intravenously (100 µg per dose, once per week). After treatment, tumors were excised, fixed in 10% buffered Zamboni solution for 24 h, paraffin-embedded, sectioned at 4 μm, and H&E-stained for subsequent imaging. The therapeutic intervention was selected based on previously reported preclinical evidence supporting the efficacy of GK-1. GK-1 has been shown to improve survival in a B16-F10 murine melanoma model (Pérez-Torres et al., 2013) and to exhibit enhanced therapeutic effects in combination with anti-PD-L1 therapy (Vera-Aguilera et al., 2017). Moreover, GK-1 has demonstrated significant anti-angiogenic activity, including reduced intratumoral vascularization and angiogenic factor expression, in a murine tumor model (Hernandez-Aceves et al., 2023). AGRO is a potential antitumor agent since it blocks nucleolin, a constitutive protein of the nucleolus that regulates the processing of primary ribosomal RNA transcripts, which participate in the regulation of the cell cycle. Cell surface-localized nucleolin is expressed in various cell types (endothelial cells and cancer cells), where it regulates differentiation, adhesion, proliferation, and angiogenesis, mediating signaling of various factors (Christian et al., 2003; Shi et al., 2007; Vivanco-Rojas et al., 2020). These studies provide the biological rationale and preclinical support for the therapeutic intervention used in the treated melanoma samples.

### 3.2 Instrument: Mueller matrix microscopy

Figure 1 shows the schematic setup of the home-improved polarimetric microscopy based on Olympus IX73 system with four Polarization-fit objective lenes (Olympus ×10, 20x 40x and 60x, with NA equal to 0.3, 0.5, 0.65 and 0.7 respectively). A solid-state LED light source (Iberoptics, Bluebox), providing illumination at wavelengths of 470, 554, 630, and 745 nm, was collimated and expanded using an aspheric condenser lens (Thorlabs, ACL5040U-DG6). Both of PSG and PSA are consist of a linear polarizer (Thorlabs, visible wire grid polarizers, WP50L-vis) and self-assemble rotated retarder (Thorlabs, Achromatic Quarte-Wave Plate, AQWP10M-580) with rotating motor (Pacific Laser Equipment, RSC-103E). Different polarized states will be generated or analyzed by rotation via Motor control (Pacific Laser Equipment, DSP-21 Motor Controller RoHs). The polarimetric signal passed through the sample to be observed, and reached a digital CMOS camera (Hamamatsu, C13440-20CU). All control signals are driven by an developed LabVIEW program. The system was thoroughly calibrated across multiple wavelengths against commercial reference polarimeters (PAX1000VIS/M and PAX1000IR1/M, Thorlabs). For both the polarization state generator (PSG) and analyzer (PSA), the calibration yielded experimental condition numbers below four with measurement stability. Further calibration details are available in Section 2.1 of the Supplementary Material.

**FIGURE 1:**
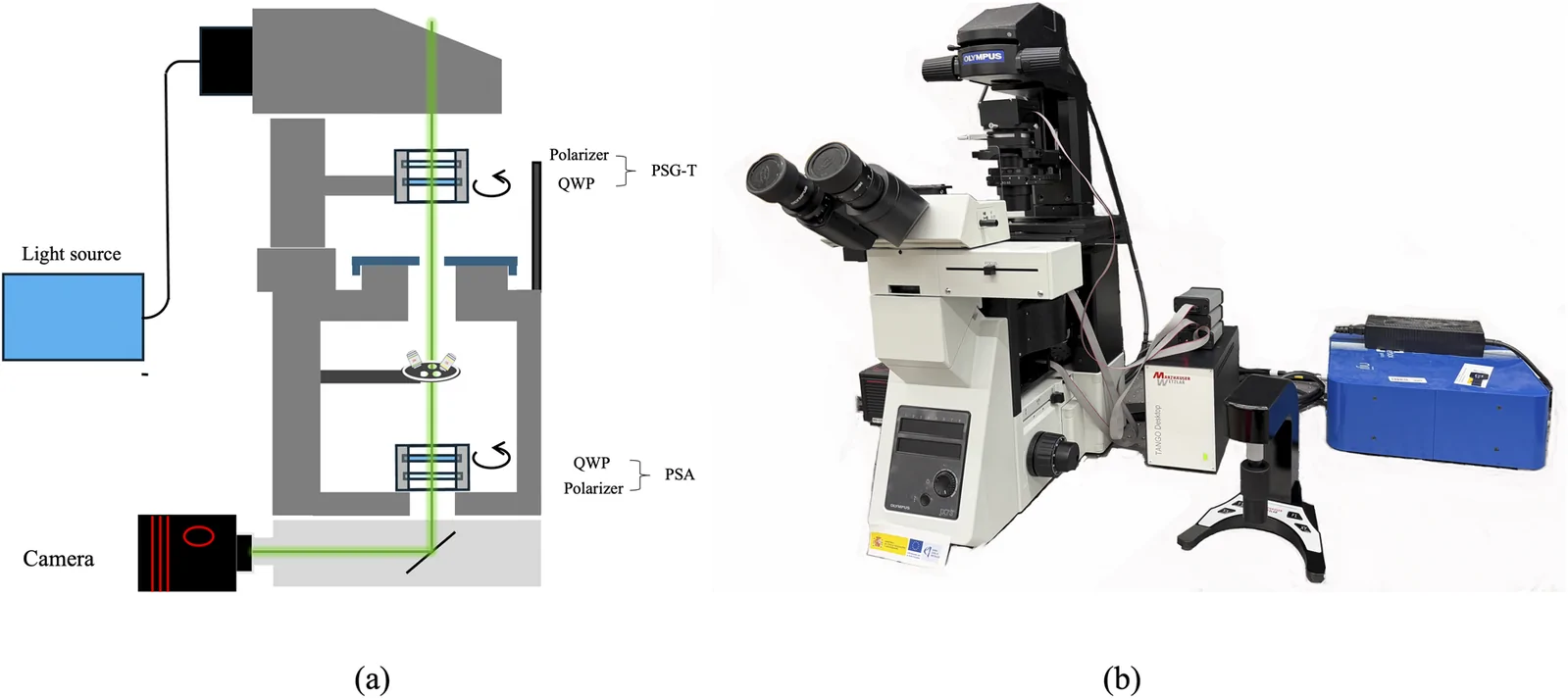
Optical architecture of the Mueller matrix microscope. **(a)** Simplified optical schematic with core components (PSG, PSA, beam splitter, and objective lens) for transmission. **(b)** Physical photograph showing the actual implementation and hardware integration of the microscopic system.

## 4 Results

For a quantitative evaluation of the proposed polarimetric computational imaging framework, eight melanoma pathology specimens—comprising four matched pairs of treatment-positive (Trt+) and treatment-negative (Trt−) tissues—were utilized in this investigation. Detailed descriptions of all samples and regions of interest (ROIs) involved in the subsequent calculations are provided in Section 2.2 of the Supplementary Material. To illustrate the processing workflow and qualitative outcomes of the proposed framework, one representative untreated–treated pair with two representative region-of-interest (ROI) were selected from H&E-stained murine melanoma specimens as typical examples: an untreated (Trt−) control (specimen I-22-249-4, B14) and a treated (Trt+) sample (specimen I-22-169-1,E5) subjected to therapeutic intervention. Pathological images of representative sample pairs, the corresponding ROIs, and the stitched fields of view are demonstrated in Figures 2a–c, while the results from the remaining cases are summarized quantitatively.

**FIGURE 2:**
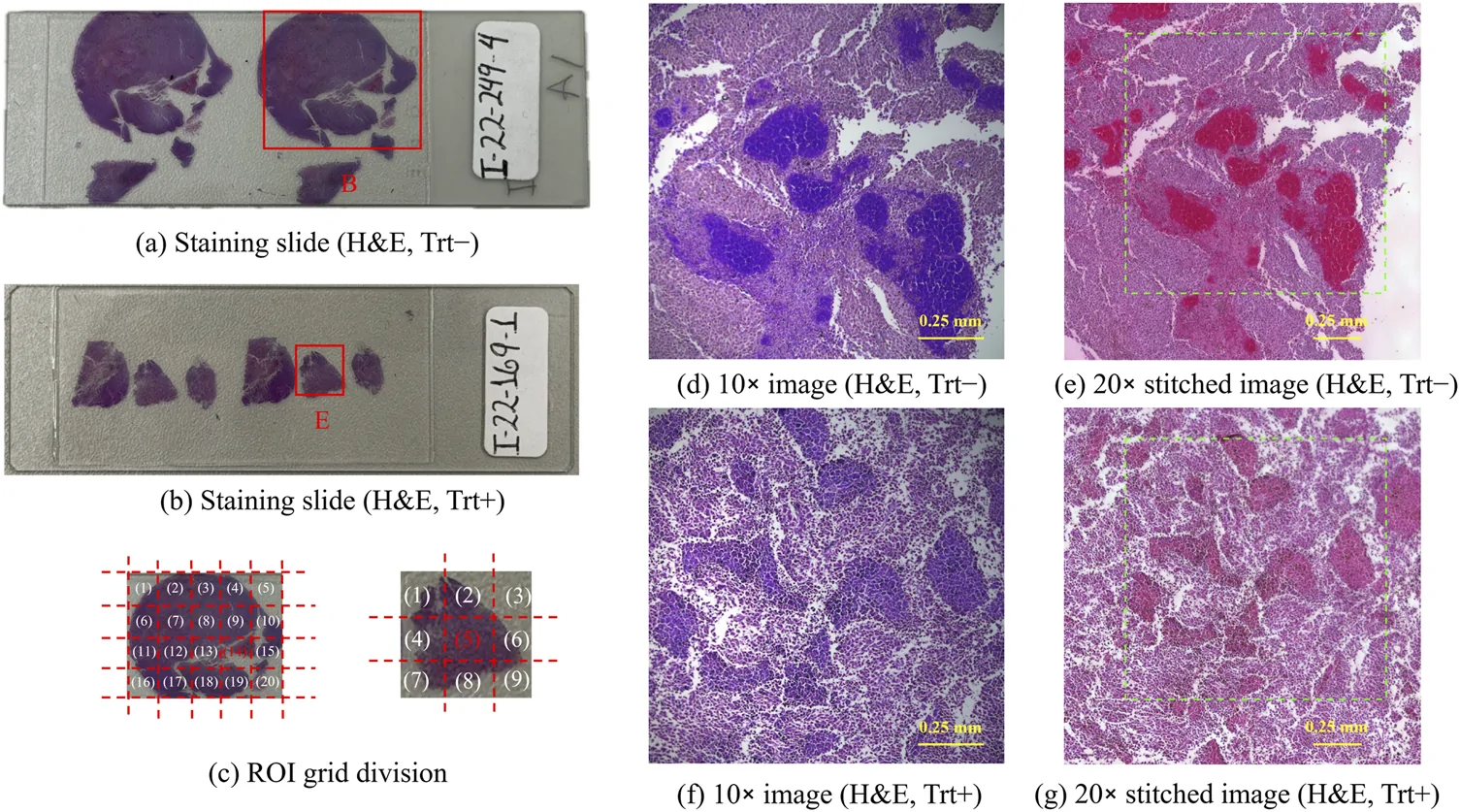
Representative demonstration of specimen selection, ROI definition, and single-FOV versus stitched $M_{00}$ RGB imaging for treated and untreated specimens. **(a)** Overview of the H&E-stained, untreated (Trt−) slide (I-22-249-4), with the region of interest **(b)** indicated. **(b)** Overview of the H&E-stained, treated (Trt+) slide (I-22-169-1), with the region of interest **(e)** indicated. **(c)** Grid-based subdivision of regions B and E into individual ROIs; ROI B14 (left, 4×5 grid) and ROI E5 (right, 3×3 grid) are highlighted in red and selected for subsequent imaging. **(d,e)** Untreated specimen (region B14): **(d)** 10× single-FOV $M_{00}$ RGB image ( 2048×2048 px); **(e)** 20× stitched panoramic $M_{00}$ RGB image ( 5638×5624 px). **(f,g)** Treated specimen (region E5): **(f)** 10× single-FOV $M_{00}$ RGB image ( 2048×2048 px); **(g)** 20× stitched panoramic $M_{00}$ RGB image ( 5638×5624 px). The resolution scale bars (yellow) for the samples are marked in the bottom-right corner of subfigures **(d–g)**, in which the 0.25-mm scale bar spans 379 pixels in the 10× image compared to 839 pixels in the 20× case. In subfigures **(e,g)**, the green dashed box marks the region corresponding to the single-FOV image in **(d)** and **(f)**, respectively.

For each selected ROI, Mueller matrix measurements were acquired at two magnifications: 10× , yielding a single FOV of 1.35 mm×1.35 mm (Figures 2d,f), and 20× , yielding tiles of 0.61 mm×0.61 mm that were scanned and stitched into a panoramic reconstruction (Figures 2e,g). Each stitched panorama was acquired over a scanning region centered on the same coordinates as the corresponding 10× FOV, spanning [x−0.55,y−0.55,x+0.35,y+0.35] mm , with a scanning step of 0.42 mm (approximately 70% of the 20× single-FOV extent) applied consistently to all specimens. The green dashed boxes in Figures 2e,g delineate the sub-regions matching the single-FOV images in Figures 2d,f, respectively. Consistent structural correspondence is observed between the single-FOV and stitched images across both treatment conditions.

### 4.1 Panoramic Mueller matrix imaging

To representatively demonstrate the Mueller matrix imaging, Figure 3 presents the polarimetric response of a sample before and after treatment, acquired under 554 nm illumination as a typical case. To provide a global view of the Mueller matrix measurements, $M_{00}$ is displayed in grayscale prior to normalization, whereas the remaining Mueller matrix elements are visualized within a fixed range of [−0.2,0.2] . This consistent visualization scheme preserves the global dynamic distribution of the measured data and enables an overall assessment of spatial variations across the entire field of view, rather than emphasizing local contrast.

**FIGURE 3:**
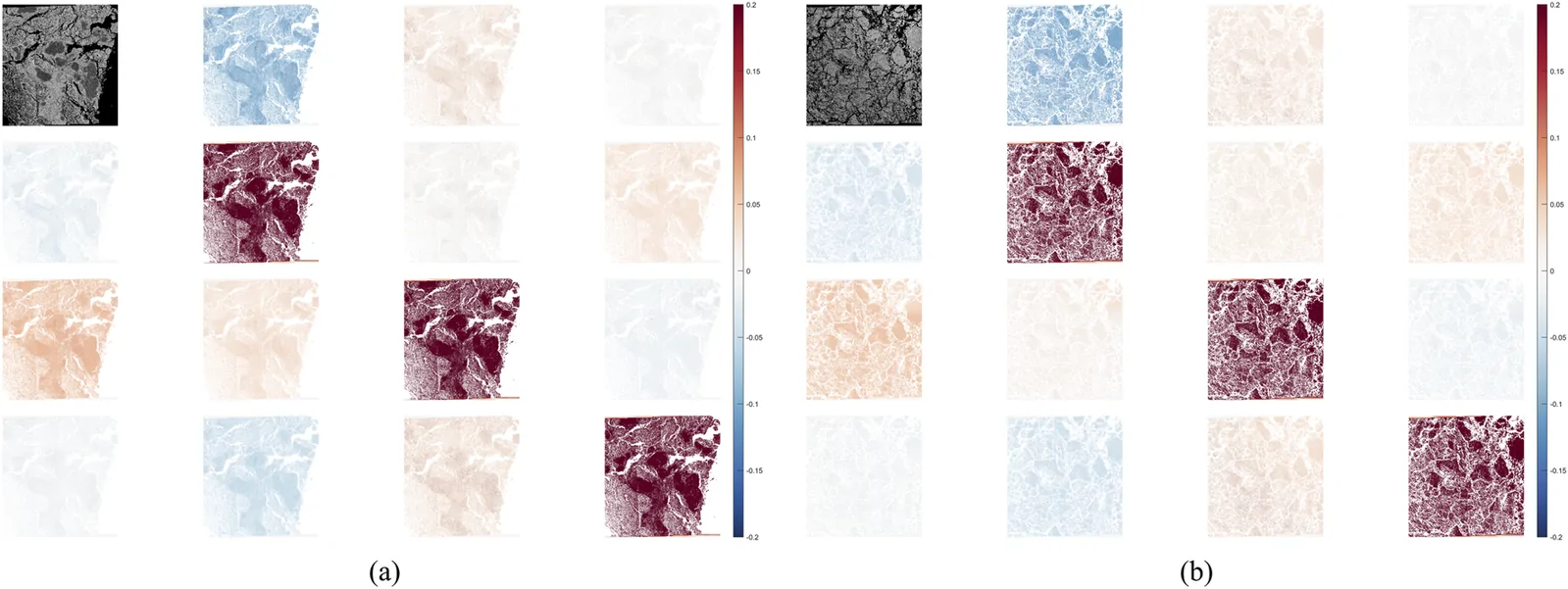
Full 4×4 Mueller matrix maps of the 20× stitched panoramas for **(a)** the untreated (Trt−) specimen and **(b)** the treated (Trt+) specimen, shown as a representative example acquired under 554 nm illumination. In each panel, $M_{00}$ is displayed as the raw intensity map, while all other elements are normalized by $M_{00}$ and shown on a common diverging colormap.

The overall polarimetric response is broadly similar between the two specimens. On one hand, the first row and column of the Mueller matrix provide information on diattenuation (selective absorption as a function of polarization) and polarizance (the sample’s capacity to polarize incident light), respectively. Although the response is weak, both of the non-treated and treated histological sections exhibit a measurable degree of linear diattenuation and polarizance, particularly along the 0° and 90° directions, on the order of 20% (relative to the full metric range), whereas the circular diattenuation and polarizance do not show significant values. This observation may be associated with anisotropic absorption in the tumor region of the mouse model, where relatively larger absolute values are observed. In contrast, within the 3×3 submatrix, the off-diagonal elements remain close to zero, whereas the diagonal elements retain relatively high values. These features are consistent with the predominantly diagonal form of the Mueller matrix observed in this specimen pair, as independently verified in Supplementary Section S1, Supplementary Figure S1, where the images are displayed using a fixed range of [−1,1] .

Therefore it is necessary to visualize $m_{11}$ , $m_{22}$ , and $m_{33}$ using an independently auto-scaled dynamic range for enhanced visualization. For a Mueller matrix with a predominantly diagonal form, the variation range of the diagonal elements is closely related to the depolarization capacity of the sample, as mathematically demonstrated in Supplementary Material, Section 1. As shown in Figure 4, this visualization reveals the spatial variations of the three diagonal elements more clearly contributed by high-resolution stitched imaging strategy (such as fine tissue scratches prominently visible in the trt− group). In addition, Beyond differences in spatial and morphological distributions, using the same color scale for both conditions enables direct comparison of the relative numerical values of the corresponding channels between the Trt+ and Trt− specimens. The diagonal elements, particularly $m_{11}$ and $m_{22}$ , are averagely smaller in the untreated specimen than in the treated specimen, which is typically indicative of a stronger depolarization effect before treatment. From a physical perspective, the enhanced depolarization may be associated with increased structural heterogeneity in the tumor tissue, including cellular disorganization, nuclear pleomorphism, and spatial variations in scattering properties.

**FIGURE 4:**
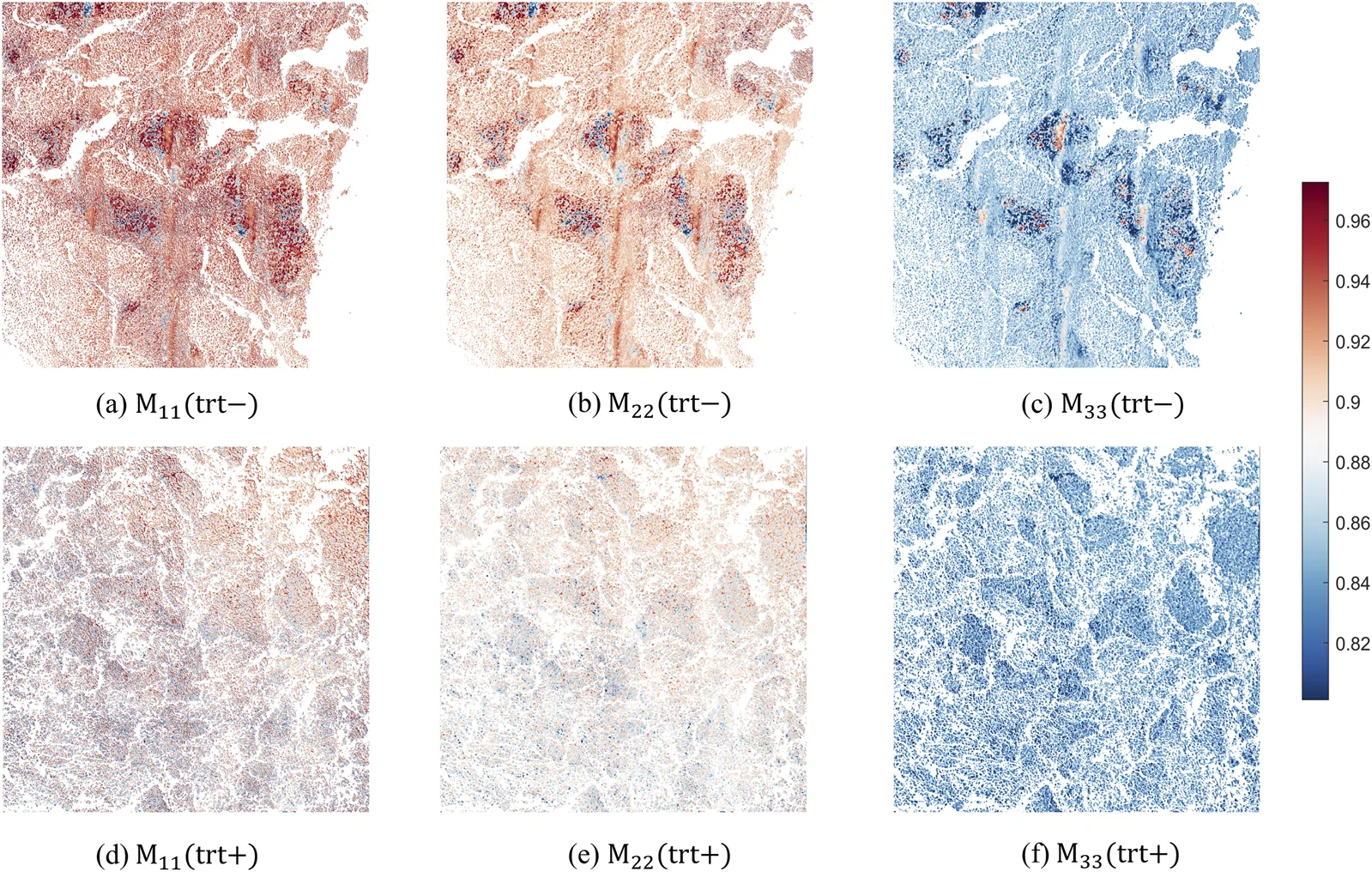
Adaptive-range visualization of normalized diagonal Mueller matrix elements ( $m_{11}$ , $m_{22}$ , $m_{33}$ ). Top row: spatial maps of $m_{11}$ , $m_{22}$ , and $m_{33}$ for a representative untreated sample (trt−) displayed on a shared colorbar range. High-resolution stitched microscopy reveals fine structural details such as tissue micro-scratches in the trt− sample (subfigure **a-c**). While bottom row (subfigure **d-f**) represent diagonal element distributions for a treated sample (trt+).

More importantly, for the diagonal-form Mueller matrices observed here, these differences are closely reflected in the spatial variation of the diagonal elements, whose dynamic range is directly related to the depolarization response, as demonstrated mathematically in Supplementary Material, Section 1. The small residual off-diagonal elements may additionally reflect weak anisotropic or birefringent contributions, together with system- and measurement-related effects. Overall, whether considering spatial heterogeneity within individual specimens or differences between the treated and untreated specimen pairs, the present results provide preliminary evidence, from both visual observation and physical interpretation, that Mueller matrix imaging can capture treatment-associated differences in the polarimetric response of melanoma tissue.

Importantly, unlike conventional histology, Mueller matrix imaging provides quantitative polarimetric observables with direct physical interpretations. Moreover, the compatibility of Mueller matrix imaging with image stitching enables high-magnification imaging while maintaining a large field of view, facilitating detailed characterization of clinically relevant tissue areas and potentially capturing the polarimetric spatial heterogeneity in untreated tumors. Together, these capabilities provide a quantitative basis for assessing tissue heterogeneity and treatment response, while motivating an intuitive visualization strategy that translates the multidimensional polarimetric observables into spatially resolved pseudo-color representations.

### 4.2 Pseudo-color mapping

In the previous discussion, we demonstrated how the stitching method performs when applied to the samples under study (Figure 2), and how polarimetric analysis, through measurement of the Mueller matrix image. In this section, we aim to go one step further by extracting the underlying physical information from these samples through the Mueller matrix decomposition and combining them with pseudo-colored approaches by applying Equation 13. Specifically, six differential-decomposition-based pseudo-color rendering methods, constructed using two combination strategies, are evaluated from two complementary perspectives: intra-mechanism mapping, which assigns the linear, orientation-resolved linear, and circular components of a single polarization-altering mechanism—dichroism, birefringence, or depolarization—to the RGB channels, and inter-mechanism mapping, which instead assigns one representative component from each of the three mechanisms to the RGB channels, grouped by component type (linear, orientation-resolved linear, or circular). First, the resulting pseudo-color representations are compared with those obtained using the Lu–Chipman decomposition, a well-established and widely adopted approach in the polarimetric community. This cross-method comparison provides a reference for assessing whether the proposed differential decomposition yields comparable polarimetric visualization for thin pathological specimens, for which the differential formulation is particularly suitable. Besides, their correspondence with H&E histology is examined to assess the histological relevance of the polarimetric features highlighted by the different pseudo-color representations.

Figure 5 lists the above-mentioned seven pseudo-color rendering methods applied to the untreated (Trt−) specimen, alongside the reference H&E-stained image (Figure 5a); the corresponding results for the treated (Trt+) specimen are shown in Figure 6 as a comparative reference, illustrating how the same set of rendering strategies performs on tissue following treatment. As depicted in the reference H&E-stained image in Figure 5a, the histologically confirmed tumor region is characterized by prominent deep-red staining. Comparative inspection across the multi-channel polarimetric pseudo-color mappings among Figure 5 b-f reveals several critical visual and structural features. First, the deep-red tumor zone identified in H&E exhibits sharp visual color differentiation from the surrounding tissue across virtually all polarimetric representation schemes, confirming the high sensitivity of polarimetric parameter combinations to pathological microstructural alterations. Second, regions displaying uniform color intensity in the H&E stain—such as the prominent red tissue mass on the right side of the field of view—exhibit marked chromatic and textural variations under different polarimetric mapping mechanisms. This divergence indicates that regions appearing relatively homogeneous in H&E can exhibit pronounced spatial heterogeneity in their polarimetric response. This observation suggests that polarimetric imaging may provide complementary sensitivity to tissue microstructural and optical heterogeneity that is not readily captured by conventional H&E contrast, motivating further physical and biophysical investigation.

**FIGURE 5:**
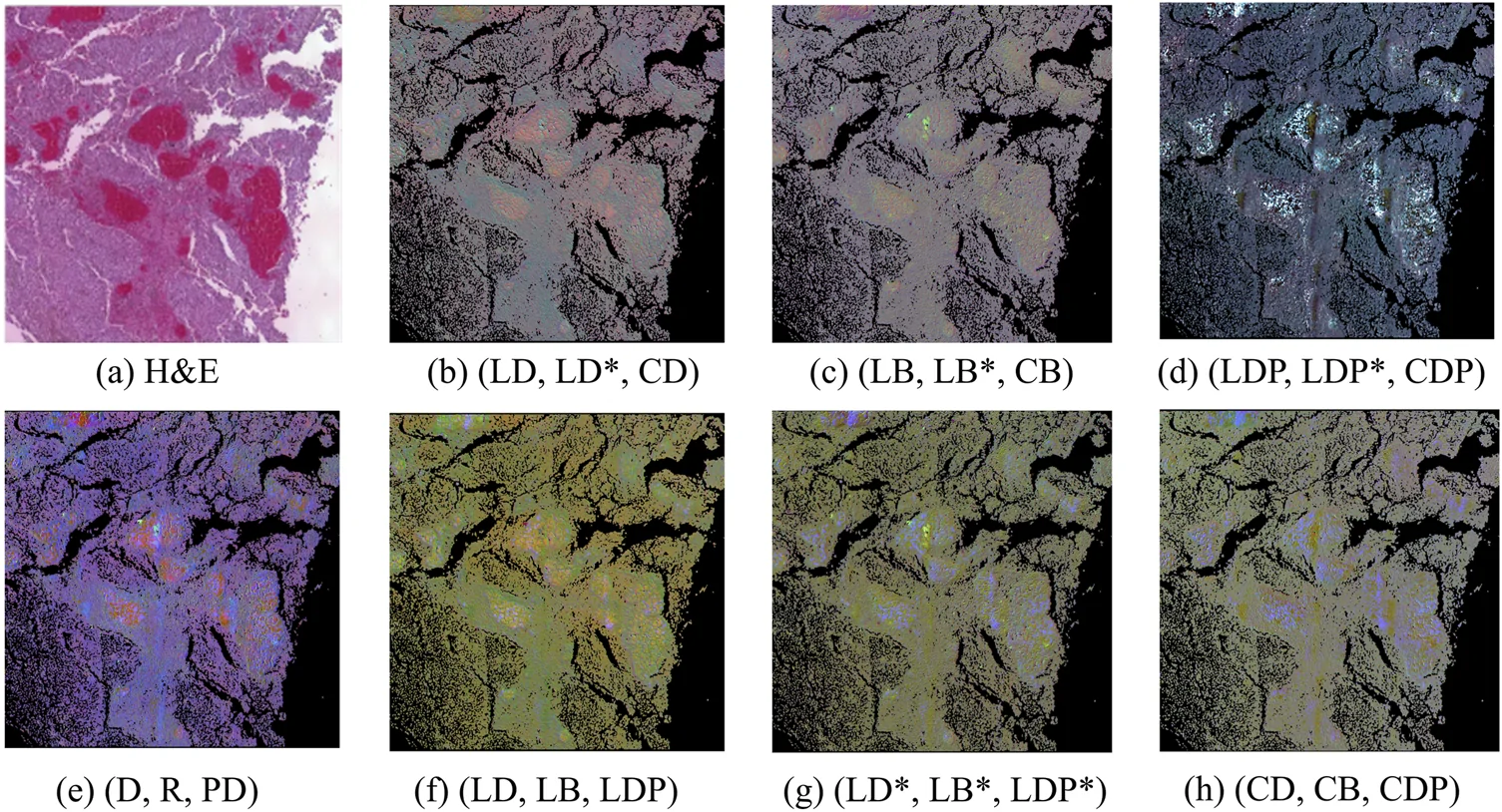
Pseudo-color visualization of polarimetric parameters for the untreated (Trt−) specimen. **(a)** Reference H&E-stained image of the corresponding tumor region. **(b–d)** Intra-mechanism mapping, assigning the linear, orientation-resolved linear, and circular components of a single mechanism to the R, G, B channels, respectively: **(b)** dichroism (LD, ${\text{LD}}^{*}$ , CD); **(c)** birefringence (LB, ${\text{LB}}^{*}$ , CB); **(d)** depolarization (LDP, ${\text{LDP}}^{*}$ , CDP). **(e)** Lu–Chipman decomposition, with diattenuation **(d)**, retardance (R), and depolarization (PD) mapped to the R, G, B channels. **(f–h)** Inter-mechanism mapping, assigning one representative component from each of the three mechanisms—dichroism, birefringence, depolarization—to the R, G, B channels, grouped by component type: **(f)** linear components (LD, LB, LDP); **(g)** orientation-resolved linear components ( ${\text{LD}}^{*}$ , ${\text{LB}}^{*}$ , ${\text{LDP}}^{*}$ ); **(h)** circular components (CD, CB, CDP).

**FIGURE 6:**
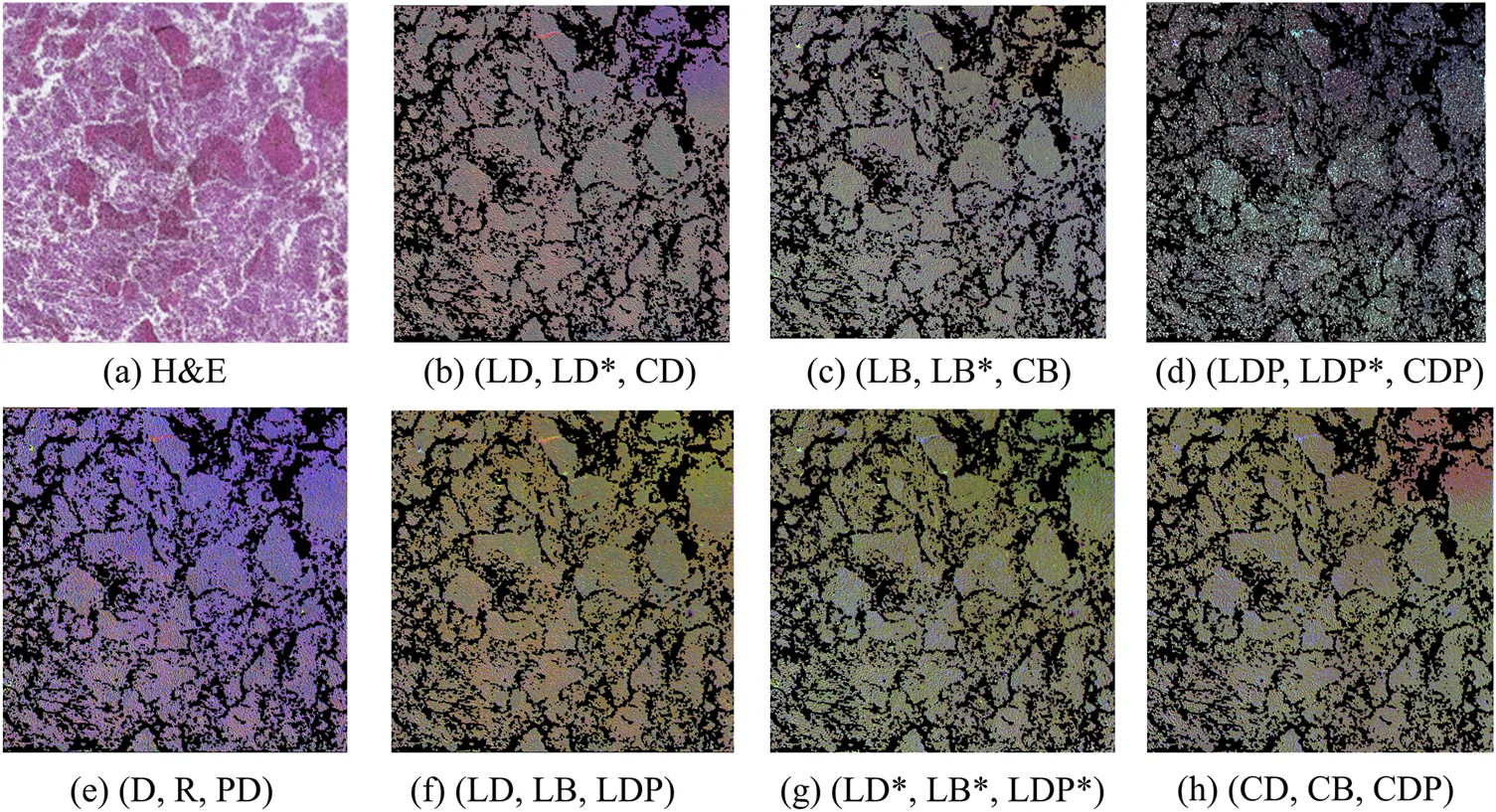
Pseudo-color visualization of polarimetric parameters for the treated (Trt+) specimen, following the same panel layout and rendering strategies as Figure 5: **(a)** reference H&E-stained image; **(b–d)** intra-mechanism mapping for dichroism, birefringence, and depolarization, respectively; **(e)** Lu–Chipman decomposition; **(f–h)** inter-mechanism mapping grouped by linear, orientation-resolved linear, and circular components, respectively.

Most notably, although all pseudo-color schemes exhibit a spatial correspondence with the tumor region identified in the H&E reference, scheme (d), which maps the differential depolarization parameters ( LDP , ${\text{LDP}}^{*}$ , and CDP ) into the RGB channels, displays a particularly distinctive visual profile. The resulting pseudo-color image exhibits a continuous spatial transition from the slate-blue background toward deep navy blue, followed by a brighter, whitish region within the tumor core. This pronounced spatial gradient indicates substantial intratumoral variation in the differential depolarization response and suggests that these parameters may provide sensitivity to spatially heterogeneous tissue properties within the tumor region.

In contrast to the untreated sample, the treated specimen (Trt+) shown in Figure 6a displays substantial tumor regression, retaining only faint, uniform pinkish staining across the tissue section. Across the majority of polarimetric mapping schemes shown in Figures 6b–g, no visually distinguishable lesion boundaries are discernible by the naked eye. This overall visual homogeneity is consistent with the post-treatment clinical diagnostic label (Trt+) and is more readily appreciated in the pseudo-color representations based on physically interpretable polarimetric parameters than in the corresponding raw Mueller matrix elements. Interestingly, scheme (d) still exhibits sparse residual contrast, manifested as isolated blue–white pixels within regions that appear weakly pinkish-purple in the corresponding H&E image. This localized correspondence suggests that the differential depolarization representation may retain sensitivity to subtle spatial variations that are less apparent in the overall H&E morphology, which highlights the potential of the differential depolarization channels to characterize subtle tissue heterogeneity beyond the dominant post-treatment appearance.

A primary advantage of polarimetric pseudo-color mapping is its intrinsic physical interpretability, in which the observed color distribution is determined by the relative contributions of physically defined polarimetric parameters rather than by optical density alone. In this sense, the pseudo-color image can be regarded as a spatial projection of the underlying polarimetric parameter space, such that both the hue and its spatial variation carry information about the local polarization response of the tissue. Taking mechanism b as an example, the tumor core exhibits a prominent orange-red hue that gradually transitions toward green at the periphery. Since LD is encoded in the Red channel and ${\text{LD}}^{*}$ in the Green channel, this spatial color transition indicates a corresponding change in the relative contribution of these two diattenuation-related responses: LD contributes more strongly within the tumor-associated region, whereas ${\text{LD}}^{*}$ becomes relatively more prominent toward the surrounding tissue. Importantly, the gradual rather than abrupt color transition suggests that the polarimetric response varies continuously across the tissue interface, providing information beyond a binary delineation of the tumor boundary. A similar physical interpretation applies to mechanism d. The bright-white appearance within the tumor region results from the simultaneous and relatively complex composition of LDP ), ${\text{LDP}}^{*}$ , and (CDP) to the three RGB channels. The resulting color therefore reflects not simply a high depolarization magnitude, but the local balance among distinct depolarization components. Moving outward from the tumor-associated region, the progressive shift toward blue corresponds to an increasing relative contribution of CDP within the selected color space. Consequently, the distinct spatial patterns generated by different pseudo-color representations may encode complementary information related to cellular organization, structural anisotropy, and tissue heterogeneity. Because these patterns originate from quantitatively defined polarimetric parameters, they provide a potential basis for linking polarimetric features with biological and pathological characteristics and for investigating their future clinical relevance.

These observations suggest that, for thin H&E-stained pathological specimens, polarimetric pseudo-color mapping not only reproduces the structural contrast conveyed by conventional H&E staining, but can further resolve regions that appear visually homogeneous under H&E into distinct physical mechanisms. While the precise biological correlates of the underlying polarimetric mechanisms remain to be fully established, this additional layer of physically informed contrast may offer clinicians a complementary perspective beyond what conventional staining alone can provide.

### 4.3 Quantitative evaluation for panoramic polarimetric imaging

As detailed in Section Equation 2.4, the evaluation conducted across different computational microscopic Mueller matrix imaging stages aims to quantitatively assess the performance of the proposed method. It should be noted that all results presented in this section are averaged across all samples to account for inter-sample variability.

#### 4.3.1 Mueller matrix image stitching

To avoid excessive detail in the main text, the evaluation indices defined above are used to quantitatively assess reconstruction fidelity, with the results summarized as within-group means for the treatment (+) and control (−) datasets. Detailed visual examples of the Mueller image stitching process are provided in Section 2.3 of the Supplementary Material. As summarized in Table 2, the quantitative evaluation demonstrates a generally high level of photometric consistency and physical fidelity across Mueller matrix elements in stitching stage.

**TABLE 2:** Quantitative evaluation of photometric discrepancy and Cross-Scale Reference MAE across the 16 raw Mueller matrix elements ( $M_{00}$ – $M_{33}$ ) for treatment (+) and control (−) specimens.

Element	Photometric mean diff. (↓)		Cross-scale ref. MAE (↓)	
	**Trt + (mean** ± **SD)**	**Trt - (mean** ± **SD)**	**Trt +**	**Trt -**
$M_{00}$	0.0300 ± 0.0323	0.0541 ± 0.0615	/	/
$M_{01}$	0.0257 ± 0.0275	0.0333 ± 0.0510	0.0451	0.0300
$M_{02}$	0.0300 ± 0.0352	0.0444 ± 0.0509	0.0407	0.0245
$M_{03}$	0.0218 ± 0.0496	0.0278 ± 0.0480	0.0201	0.0111
$M_{10}$	0.0161 ± 0.0369	0.0554 ± 0.0526	0.0211	0.0206
$M_{11}$	0.0307 ± 0.0327	0.0522 ± 0.0623	0.0475	0.0399
$M_{12}$	0.0329 ± 0.0299	0.0482 ± 0.0405	0.0168	0.0173
$M_{13}$	0.0451 ± 0.0737	0.0773 ± 0.0581	0.0098	0.0102
$M_{20}$	0.0269 ± 0.0338	0.0546 ± 0.0563	0.0240	0.0085
$M_{21}$	0.0426 ± 0.0263	0.0427 ± 0.0504	0.0208	0.0109
$M_{22}$	0.0306 ± 0.0329	0.0543 ± 0.0623	0.0272	0.0275
$M_{23}$	0.0525 ± 0.1423	0.0613 ± 0.0565	0.0094	0.0095
$M_{30}$	0.0210 ± 0.0455	0.0401 ± 0.0511	0.0226	0.0115
$M_{31}$	0.0388 ± 0.0451	0.0452 ± 0.0539	0.0179	0.0128
$M_{32}$	0.0531 ± 0.0561	0.0639 ± 0.0529	0.0498	0.0277
$M_{33}$	0.0301 ± 0.0329	0.0519 ± 0.0627	0.0274	0.0329

**TABLE 3:** Quantitative comparison of textural and information-encoding quality across pseudo-color rendering strategies, for untreated (Trt−) and treated (Trt+) specimens. B–D: intra-mechanism; E: Lu–Chipman reference; F–H: inter-mechanism. Values are Mean. Best in **bold**, second-best underlined.

Condition	Strategy	**GLCM contrast** ↑	**Image entropy** ↑	**AG** ↑
Trt−	B (dichroism, intra)	0.8256	5.4411	0.0460
	C (birefringence, intra)	1.0699	5.7285	0.0563
	D (depolarization, intra)	0.9510	**5.8888**	0.0518
	*E (Lu–Chipman)*	1.0903	5.7368	0.0562
	F (linear, inter)	0.6334	5.1483	0.0367
	G (orient.-lin., inter)	1.1171	5.8087	**0.0591**
	H (circular, inter)	**1.1476**	5.6625	0.0550
Trt+	B (dichroism, intra)	1.0029	4.9830	0.0486
	C (birefringence, intra)	1.1231	5.1504	0.0539
	D (depolarization, intra)	1.0467	**5.5280**	**0.0570**
	E (Lu–Chipman)	**1.2393**	5.2886	**0.0570**
	F (linear, inter)	0.7731	4.5787	0.0385
	G (orient.-lin., inter)	1.1443	5.2385	0.0554
	H (circular, inter)	1.1145	5.1168	0.0540

Value in bold means it is the best considering the viusal effect.

The photometric discrepancy quantifies defined in Equation 14 the absolute photometric discrepancy across tile interfaces locally, providing a quantitative measure of the registration and stitching accuracy achieved by the SIFT-based approach. This average incorporates both structural variations in the stitched configurations, ranging from multiple tile arrays to individual tiles, and biological variability across four distinct samples within each category. The value of photometric discrepancy remains relatively small across the evaluated elements, with the largest mean discrepancy reaching only 5.25%. Globally, the Cross-Scale Reference MAE calculated through Equation 15 further provides complementary evidence of the fidelity of the reconstructed data after stitching, with rather low absolute errors (2.5% at maximum considering that the Mueller matrix elements are normalized within the range [−1,1] ) observed for the all normalized Mueller matrix elements.

#### 4.3.2 Polarimetric pseudo-color imaging

While the preceding analysis qualitatively assessed the visual enhancement provided by the pseudo-color representation using representative sample pairs, this section extends the evaluation to a systematic comparison of different pseudo-color methods. Using the visual operators defined in Equations 16–18, Section 2.4, we quantitatively assess their relative capability to enhance the histological appearance to different degrees, particularly in terms of textural features, information content, and image contrast across the evaluated samples.

Quantitatively, no single pseudo-color strategy consistently maximizes all three visual enhancement metrics, highlighting the complementary nature of the evaluated representations as evaluation indexes selected among multiple samples summarized in Table 3. Scheme D achieves the highest image entropy for both untreated and treated specimens (5.8888 and 5.5280, respectively), indicating a consistently information-rich color representation associated with the combined encoding of LDP , ${\text{LDP}}^{*}$ , and CDP . Scheme G provides a comparatively balanced performance across the three metrics and achieves the highest average gradient for the untreated specimen (0.0591), suggesting pronounced local spatial variation. Notably, the Lu–Chipman-based scheme E yields the highest GLCM contrast for the treated specimen (1.2393) and is tied with scheme D for the highest average gradient (0.0570), demonstrating that the conventional decomposition can also provide strong contrast and spatial delineation. These results therefore do not indicate a universal superiority of one representation, but rather suggest that different decomposition and channel-combination strategies emphasize complementary aspects of polarimetric image structure.

Importantly, the differential-decomposition-based representations exhibit overall visual performance comparable to that of the Lu–Chipman-based representation, from both qualitative and quantitative perspectives. Qualitatively, the differential and Lu–Chipman schemes produce similar levels of tissue contrast and spatial delineation, with both approaches clearly highlighting the major tissue regions while exhibiting distinct, decomposition-dependent color patterns. Quantitatively, their performance is likewise comparable across the three visual enhancement metrics. In particular, the Lu–Chipman-based scheme E achieves the highest GLCM contrast for the treated specimen and a similarly high average gradient to scheme D, whereas the differential schemes, particularly scheme D, provide higher image entropy. These complementary metric profiles indicate that the differential decomposition can achieve a visual representation comparable to the established Lu–Chipman decomposition for thin pathologic tissue sections. Honestly, the enhancement varies among different types of samples, all the polarimetric pseudo-coating methods offer a physically interpretable encoding.

Establishing a robust relationship between the proposed polarimetric imaging approach and its potential relevance to diagnostic histopathological imaging requires evaluation on larger sample cohorts and from multiple complementary perspectives. Therefore, as summarized in Table 4, we further examine whether the visually enhanced regions identified from the polarimetric pseudo-color representation exhibit spatial correspondence with histologically defined tissue regions. Specifically, as described in Section 2.4 and detailed in Section 2.4 of supplymentary material, we focus exclusively on the untreated (Trt-) samples, comparing the tumor regions identified from H&E staining with extracted structural mask from pseudo-coloring maps. The spatial correspondence between these regions and the H&E-derived tumor regions is then quantified using the Dice Similarity Coefficient (DSC), a commonly used metric for assessing spatial overlap in medical image segmentation with it defintion given in Equation 19.

**TABLE 4:** Cross-Modality Comparision between H&E Histology Reference and Various Polarimetric Virtual Staining Mapping Strategies (B–H) for Untreated Specimens (Trt−) . Metric is Macro-Regional Overlap (DSC). Best in **bold**, second-best underlined.

Polarimetric staining strategy	Macro-overlap: DSC (↑)
B (dichroism, intra)	0.5198
C (birefringence, intra)	0.5063
D (depolarization, intra)	**0.5971**
E (*Lu–Chipman*)	0.5725
F (linear, inter)	0.5253
G (orientation-resolved linear, inter)	0.5214
H (circular, inter)	0.5424

Value in bold means it is the best considering the viusal effect.

The DSC results provide an initial quantitative assessment of the spatial correspondence between the polarimetric pseudo-color representations and the H&E-defined pathological regions. Among the evaluated mapping strategies, Strategy D (depolarization, intra) achieved the highest DSC of 0.5971, indicating that depolarization-derived polarimetric features provided the strongest spatial correspondence with the histologically identified tissue regions. Strategy E (*Lu–Chipman*) showed the second-highest performance, with a DSC of 0.5725, further supporting the relevance of depolarization-related information for highlighting histologically distinct structures. The remaining mapping schemes (B, C, F, G, and H) exhibited moderate spatial overlap, with DSC values ranging from 0.5063 to 0.5424.

Overall, these results provide preliminary quantitative evidence that depolarization-informed pseudo-color representations may offer a more effective visual correspondence to histologically distinct tissue structures than the other evaluated mapping schemes. It should be emphasized, however, that this analysis represents an initial attempt to establish such cross-modality correspondence. In particular, the SLIC-based feature extraction employed here was not specifically optimized for polarimetric pseudo-color representations, leaving considerable room for further improvement through modality-specific feature extraction and segmentation strategies.

Taken together, the detailed quantitative evaluation demonstrates the potential of the proposed framework at both the methodological and imaging levels. First, the low reconstruction and registration errors achieved by the stitching framework indicate its stability in extending polarimetric imaging from individual fields of view to large-area acquisitions, while maintaining spatial consistency and preserving the physical interpretability of the reconstructed polarimetric information. Second, the resulting large-FOV polarimetric mappings provide a more spatially continuous, spatially distinguishable, and physically interpretable representation of tissue structural variations than direct visualization of individual Mueller matrix elements. The preliminary quantitative correspondence observed between these polarimetric representations and H&E-defined tissue features further suggests that the spatially resolved polarimetric contrasts may capture structural characteristics relevant to conventional histological assessment. Nevertheless, establishing their broader biological and clinical relevance will require further validation from a more medically oriented perspective, including comparisons with clinically relevant histopathological features and evaluation across a substantially larger and more diverse cohort of samples.

## 5 Conclusion

In this study, we presented a physics-informed computational framework, termed Computational Mueller Matrix Microscopic Pathology, integrating differential Mueller matrix decomposition, wide-field image stitching, and pseudo-color visualization into a unified workflow for quantitative tissue characterization. Applied to histological murine melanoma specimens under untreated (Trt−) and treated (Trt+) conditions, the framework enabled physically interpretable visualization of the underlying polarimetric mechanisms while preserving both high spatial resolution and large field of view through image stitching.

For the thin pathological sections investigated in this study, the differential decomposition approaches and the Lu–Chipman decomposition provided comparable representations of tissue structures, with consistent preservation of local structural variations and tissue heterogeneity. More importantly, the resulting mechanism-specific pseudo-color representations provide a physically interpretable means of visualizing polarimetric information: different color components can be associated with distinct optical mechanisms, including dichroism, birefringence, and depolarization, rather than merely representing intensity or arbitrary image contrast. This physical interpretability facilitates the interpretation of tissue-related polarimetric patterns by linking their spatial characteristics to the underlying optical responses and to established histopathological knowledge, with guidance from experienced medical experts.

Within these mechanism-specific contrasts, depolarization emerged as a particularly notable direction in the melanoma samples. This observation was supported consistently across multiple aspects of the analysis, including the numerical variations of the Mueller matrix elements, the mean values of the derived polarimetric parameters, the spatial heterogeneity of the corresponding maps, and their preliminary spatial correspondence with H&E-defined tissue regions. In particular, untreated tumors exhibited a broader and more heterogeneous depolarization response, whereas treated tumors showed a comparatively more localized distribution. The convergence of these quantitative and spatial observations suggests that depolarization is not merely a visually distinctive contrast, but may be related to structural heterogeneity within the tumor tissue and its treatment-associated modification. While this preliminary observation does not establish a direct biological or causal relationship, it highlights depolarization as a potentially informative polarimetric feature for further investigation of melanoma-associated tissue structure and treatment response.

More broadly, this work highlights the potential of Mueller matrix polarimetry to bridge conventional histological observations and quantitative physical tissue descriptors. By combining large-field imaging, physically grounded parameter extraction, and intuitive visualization, the proposed framework provides a robust foundation for future computational pathology studies, biomarker discovery, and treatment-monitoring applications. Future work will focus on larger pathologist-annotated cohorts and on integrating polarimetric biomarkers with machine-learning approaches for automated tissue characterization and treatment-response prediction.

## Data Availability

The original contributions presented in the study are included in the article/Supplementary Material, further inquiries can be directed to the corresponding author.
