# Supplementary material for: Computational Mueller matrix microscopic pathology based on differential decomposition for medical reference mapping
Source: Front Bioinform. 2026 Oct 26;6:1953765. doi: 10.3389/fbinf.2026.1953765 (PMC13745666; doi:10.3389/fbinf.2026.1953765)
Supplement: Supplementary file 1 [file Presentation1.pdf]

Supplementary Material

1 DEPOLARIZATION BOUNDS FOR DIAGONAL MUELLER MATRICES

Depolarization represents a crucial optical property when characterizing complex biological media from a polarization perspective. In polarimetric analysis, a wide range of depolarizing tissue specimens—particularly pathological tissue samples exhibiting structural heterogeneity—can be well approximated by a pixel-wise normalized diagonal depolarizing Mueller matrix:

$$\mathbf{m} \approx \text{diag}(1, a, b, c) \tag{S1}$$

where $a, b, c \in [-1, 1]$ denote the normalized diagonal matrix elements corresponding to the principal depolarization factors along orthogonal polarization axes, normalized pixel-wise by the total intensity element $M_{0,0}$. For example, normalized Mueller matrix of two representative samples presented in the main text are illustrated here in Fig S1 when we set the scale from -1 to 1:

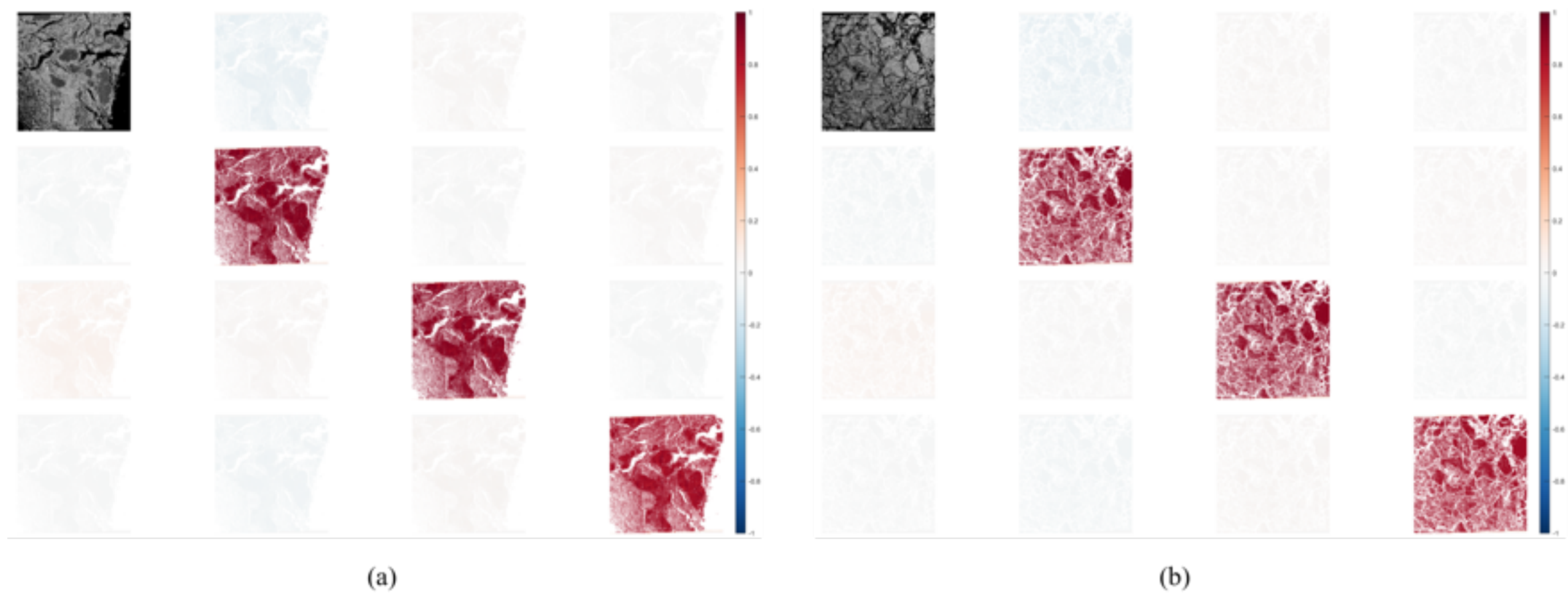

Figure S1. Full 4×4 Mueller matrix maps of the $20\times$ stitched panoramas for (a) the untreated (Trt$-$) specimen and (b) the treated (Trt+) specimen, shown as a representative example acquired under 554 nm illumination, with the common range from -1 to 1.

From a conceptual standpoint, the fluctuation range of normalized Mueller matrix elements determines the empirical data distribution, thereby dictating the polarization characterization capability (e.g., depolarization performance). A rigorous mathematical demonstration validating this perspective will be presented in the following section. Note that the following theoretical analysis specifically applies to media exhibiting this diagonal depolarizing behavior. Strating from global depolarization, using the definition proposed by Gil and BernabeuGil and Bernabeu (1985, 1986), the depolarization index P_Δ of a Mueller matrix M is defined as:

$$P_\Delta = \frac{\left(\sum_{i,j=0}^{3} \mathbf{M}_{i,j}^2 - \mathbf{M}_{0,0}^2\right)^{1/2}}{\sqrt{3}\mathbf{M}_{0,0}} \tag{S2}$$

For the normalized diagonal Mueller matrix $\mathbf{m} = \mathrm{diag}(1, a, b, c)$, with $M_{0,0} = 1$, the sum of the squared elements simplifies directly to $\sum_{i,j=0}^{3} m_{i,j}^2 = 1 + a^2 + b^2 + c^2$. Substituting these terms into Eq. (2) yields:

$$P_\Delta = \frac{\sqrt{(1 + a^2 + b^2 + c^2) - 1}}{\sqrt{3}} = \sqrt{\frac{a^2 + b^2 + c^2}{3}} \tag{S3}$$

To evaluate how local variations in individual diagonal elements mathematically constrain the overall depolarization strength, we define $m_{\min} = \min(a, b, c)$. Since $a^2 + b^2 + c^2 \geq 3m_{\min}^2$, we establish the analytical lower bound on P_Δ:

$$P_\Delta = \sqrt{\frac{a^2 + b^2 + c^2}{3}} \geq \sqrt{\frac{3 \cdot m_{\min}^2}{3}} = m_{\min} \tag{S4}$$

Therefore, analyzing the range and minimal values of normalized diagonal elements provides a mathematically rigorous metric directly anchored to global depolarization strength. Similarly, the differential-based depolarization metrics are extracted as Section 2.2. For the normalized diagonal depolarizing Mueller matrix $\mathbf{m} = \mathrm{diag}(1, a, b, c)$, the differential matrix $\mathbf{L} = \ln(\mathbf{m})$ can be directly calculated as $\mathbf{L} = \mathrm{diag}(0, \ln(a), \ln(b), \ln(c))$. According to the algebraic structure of the symmetric component $\mathbf{L}_u$, which characterizes the depolarizing behavior, the linear and circular depolarization rates (LDP, LDP', and CDP) are defined as the negative values of the logarithmic diagonal elements:

$$LDP = -\ln(a), \quad LDP' = -\ln(b), \quad CDP = -\ln(c) \tag{S5}$$

According to the intrinsic properties of the function $-\ln(x)$, particularly for transmission measurements where the principal depolarization factors $a, b, c \in [0, 1]$, $-\ln(x)$ acts as a monotonically decreasing function within the interval $(0, 1]$. As $x \to 0^+$, $-\ln(x) \to +\infty$, and as $x = 1$, $-\ln(x) = 0$, yielding a value range of $[0, +\infty)$. This mathematical behavior indicates that the minimum diagonal element $\min(a, b, c)$ logarithmically governs the differential depolarization rates (LDP, LDP', CDP). Consequently, a smaller $\min(a, b, c)$ (i.e., approaching 0) induces a disproportionately large depolarization rate (approaching $+\infty$), whereas values approaching unity drive the rates toward 0. Therefore, the effective range and specifically the lower bound of $[a, b, c]$ are profoundly correlated with the differential depolarization properties, providing a logarithmically scaled metric for evaluating the medium's depolarizing strength.

2 EXPERIMENTAL DETAILS

2.1 Instrument Calibration

In our setup, the Polarization State Generator (PSG) and Polarization State Analyzer (PSA) were both designed to generate and analyze different distinct polarization states to realize $N_g \times N_a$ pair measurement in time-division style by using the a rotated quarter-waveplate (QWP) after a linear polarizerSmith (2002).

During the calibration process, discrete rotation angles were swept to thoroughly map the manifold of achievable polarization states on the Poincaré sphere. Specifically, the generated polarization states of the PSG were characterized using a commercial polarimeter, whereas the state vectors for the PSA were retrieved from air measurements (empty-system reference) using Eq. (S6).

$$A = I_{\text{air}} W^{-1} \tag{S6}$$

where I_{air} denotes the measured intensity matrix for air, and G represents the calibrated generator matrix of the PSG.

To optimize the performance and maintain the robustness of Mueller matrix polarimetric system across the working spectrum, an optimized subset of six states ($N_g = N_a = 6$) was selected by minimizing the condition number (CN) of the Generator matrix W and Analyzer matrix A. The generated and analyzed polarization states were represented in Poincaré sphere, as illustrated in Fig. S2. The top and bottom rows display the normalized Stokes state trajectories of PSG (W) and PSA (A), respectively, across four operating wavelengths (470 nm, 554 nm, 630 nm, and 745 nm).

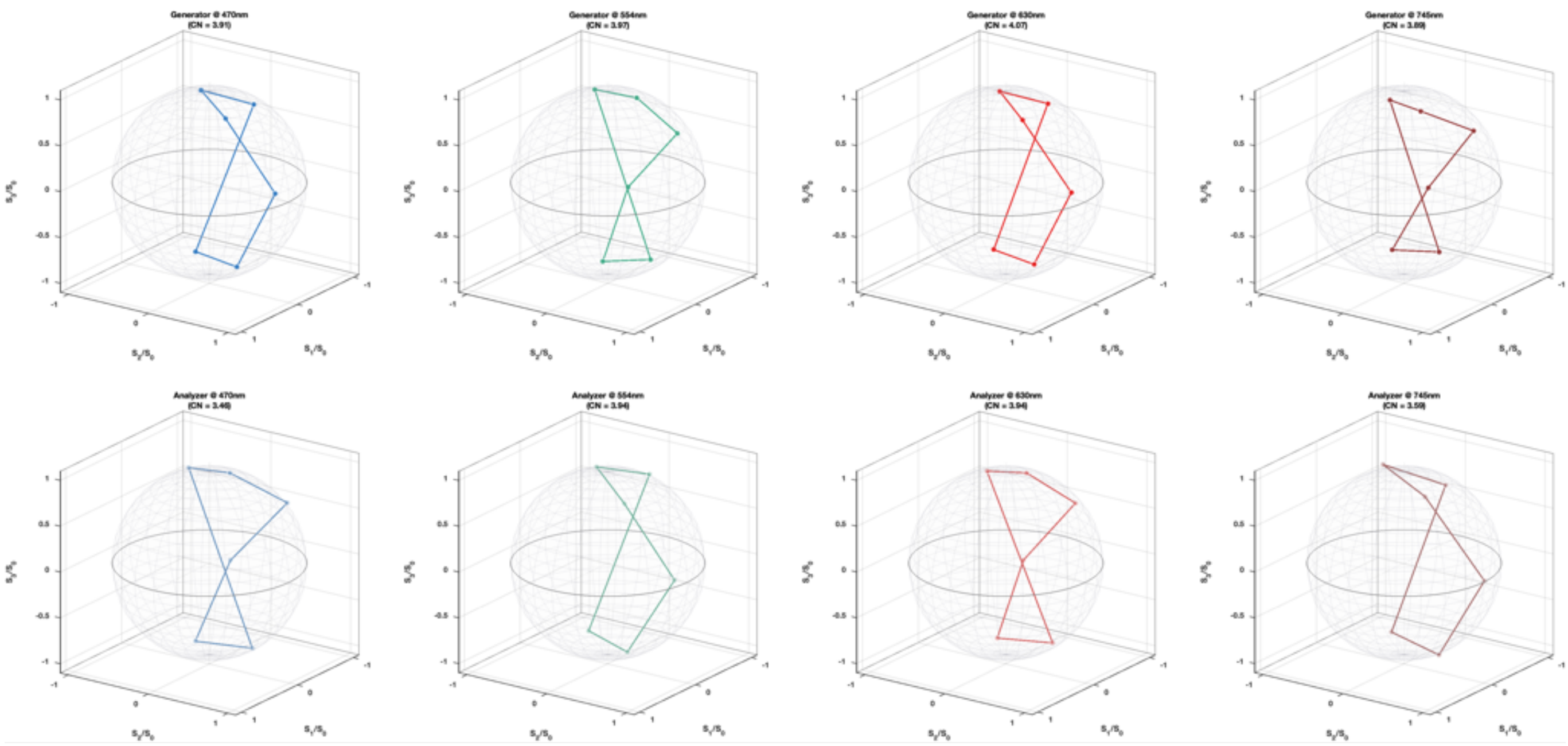

Figure S2. Polarization state distributions on the Poincaré sphere and condition numbers (CN) for the transmission Mueller matrix polarimeter. The top row depicts the polarization state generator (PSG) matrices (W) and the bottom row illustrates the polarization state analyzer (PSA) matrices (A) across four operational wavelengths: 470 nm (blue), 554 nm (green), 630 nm (red), and 745 nm (red-brown). Solid circles represent the normalized Stokes vectors ($S_1/S_0, S_2/S_0, S_3/S_0$) forming closed spherical trajectories. Low condition numbers (CN $\approx 3.48 - 4.07$) across all the used wavelengths demonstrate optimal calibration.

Due to the physical constraint of utilizing a rotating quarter-wave plate (QWP) paired with a fixed polarizer—rather than dual Liquid Crystal Variable Retarders (LCVRs)—the generated states traverse a specific trajectory along the sphere. Consequently, the condition numbers cannot reach the theoretical lower bound of a fully spherical optimization. Nevertheless, the calculated condition numbers (CN) remain low and stable enough across all spectral channels, ranging from 3.88 to 4.07 for the generator matrices and from 3.48 to 3.94 for the analyzer matrices. Crucially, adopting a rotating QWP architecture offers distinct mechanical and optical advantages over LCVR-based setups. While LCVR systems require exceptional

spatial uniformity and are highly prone to spatially non-uniform retardance responses—which frequently introduce artifacts in polarimetric imaging (especially important for microscopic imaging) and accumulate during Mueller matrix reconstruction. Furthermore, it possesses superior thermal immunity and long-term mechanical repeatability. The preceding discussion and calibration results amply demonstrate the soundness of the system design and the robustness of the measurements.

2.2 Samples and ROI selection

The analysis and conclusions presented in this work are supported by measurements obtained from eight representative samples, comprising four untreated (Trt-) samples and four treated (Trt+) samples as a control, from which different regions of interest (ROIs) were evaluated. The ROIs were selected to encompass a broad range of visually and morphologically distinct histopathological features, thereby capturing the intrinsic structural heterogeneity present across primary tumor specimens. A representative pair of examples (Trt+ vs Trt-) was presented in the main text, while the remaining samples and ROIs were included in the evaluation index calculations. Although the overall number of biological specimens remains limited compared with large-scale data-driven studies, the experimental evaluation was extended across these additional tumor samples and morphologically distinct histopathological regions to examine the robustness and consistency of the proposed framework under varying tissue architectures and sample conditions (treated and untreated mice). The results therefore provide supporting evidence that the proposed approach remains applicable beyond the representative examples shown in the main text, while a larger cohort-level study will be required for comprehensive statistical validation of treatment-associated differences.

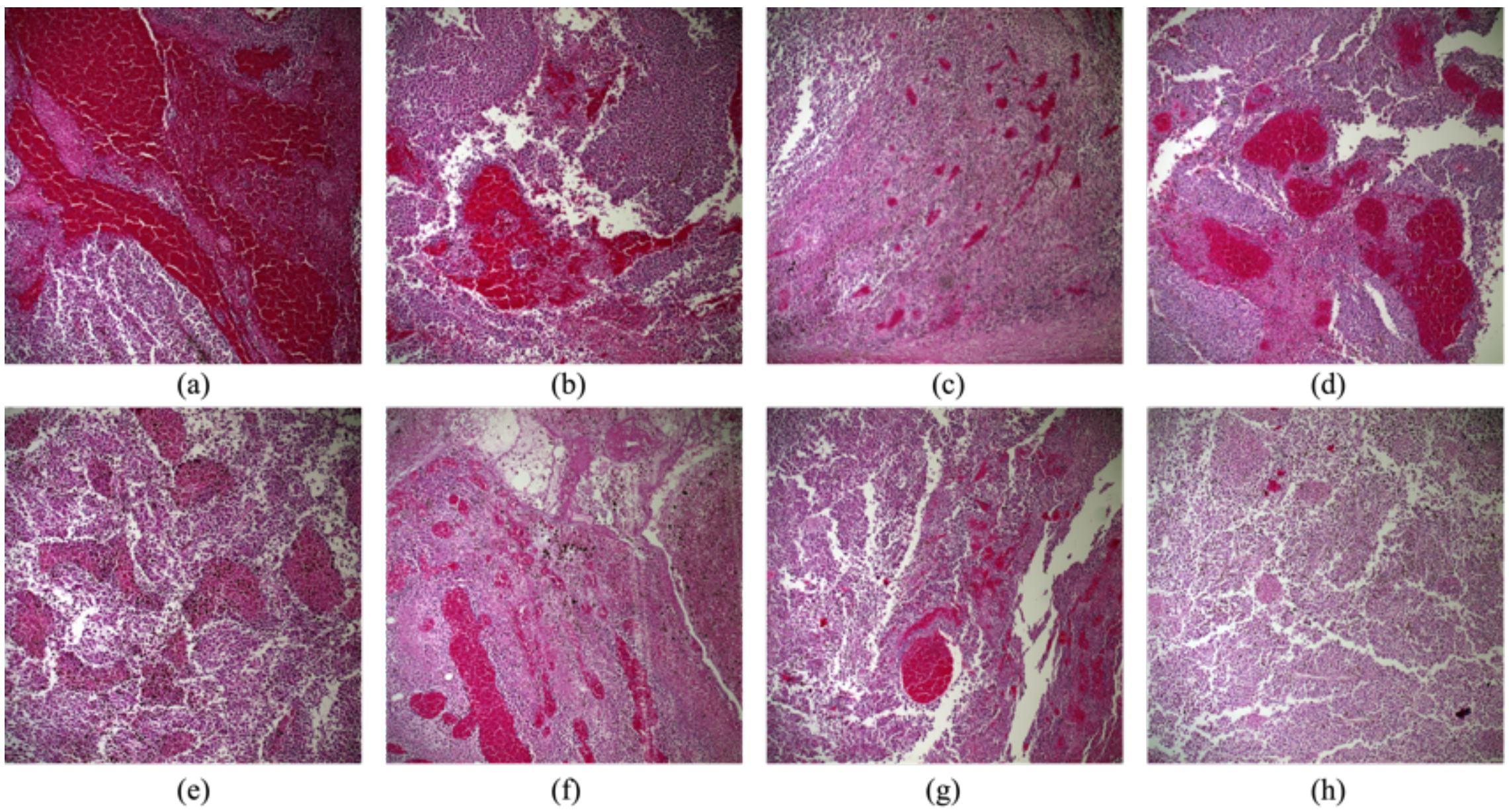

Figure S3. H&E-stained histological images of all eight melanoma regions of interest (ROIs) analyzed in this study across treated (Trt^{+}) and untreated (Trt^{-}) groups. Images were acquired under a $10\times$ objective lens within a single field of view (FOV) to capture pathological heterogeneity across the dataset. **(a–d) Untreated group (Trt^{-}):** Sample label (a) I-22-153-5(B), (b) I-22-153-5(C), (c) I-22-255-10(A), and (d) I-22-249-4(B). **(e–h) Treated group (Trt^{+}):** Sample label (e) I-22-169-1(E), (f) I-22-176-8(A), (g) I-22-176-8(D), and (h) I-22-178-10(A).

To evaluate the structural features across the experimental cohort, all eight H&E-stained RGB images analyzed in this study were captured using a $10\times$ objective lens with its single FOV (Fig. S3). From a purely visual perspective, marked morphological and staining or chromatic differences are apparent between primary tumor of the untreated (Trt-) and treated (Trt+) mice. The untreated group (Trt-, Fig. S3a–d) is characterized by extensive, continuous regions of intense deep-red staining interspersed with densely packed purple-stained cellular areas. In H&E tumor sections, these deep-red or eosinophilic regions are consistent to numerous and different-caliber congested blood vessels and probably hemorrhagic areas. Densely basophilic purple regions indicate high nuclear density belonging to tumor cellsChan (2014). In contrast, the treated group (Trt+, Fig. S3e–h) exhibits a visually lighter and more heterogeneous appearance, with fewer extensive blood vessels (eosinophilic deep-red regions) and broader pale-pink, nonfibrous, or unstained extracellular matrix (ECM) areas that limit tumor cell clusters. Collectively, these features are visually consistent with substantial treatment-associated alterations of the tumor microenvironment (ECM organization, angiogenesis and tumor cells architecture) Aung et al. (2017); Stein et al. (2019). Mice with melanoma were treated with the GK-1 peptide and the aptamer AGRO, both of which have been shown to have antiangiogenic effectsHernandez-Aceves et al. (2023); Vivanco-Rojas et al. (2020).

Intra-group variations are also evident among the selected specimens. Within the untreated (Trt^{-}) group, images (a) and (b) (sample I-22-153-5(B) and I-22-153-5(C)) contain prominent coalescent deep-red regions (congestive blood vessels) surrounded by densely purple-stained tissue (densely packed tumor cells), producing a highly heterogeneous appearance. In fact, the heterogeneity of the tumor microenvironment is a characteristic of neoplasms. By comparison, subfig (c) (sample I-22-255-10(A)) exhibits a more spatially uniform fine-grained purple texture with relatively limited deep-red staining, indicating a more homogeneous cellular distribution at the imaged scale.

For the treated (Trt^{+}) set, subfigs (f) and (g) (sample IDs I-22-176-8(A) and I-22-176-8(D)) retain localized clusters of deep-red and densely purple-stained regions embedded within broader pale stromal areas, suggesting that the antiangiogenic effect of GK-1 and AGRO, and probably other antitumor effects demonstrated for the GK-1 peptide, caused a remodeling of the tumor microenvironment, leaving some areas of heterogeneous residual tissue organization. In contrast, image (h) (sample I-22-178-10(A)) displays a diffuse, low-contrast, web-like cellular pattern distributed across a broad pale background, representing a markedly different tumoral tissue architecture from the densely packed appearance observed in several untreated specimens. These intra-group differences highlight the substantial morphological heterogeneity present within both cohorts.

2.3 Mueller matrix Stitching Experimental Details

To achieve a field of view (FOV) comparable to that of a lower-magnification ($10\times$) objective while preserving the high spatial resolution of a $20\times$ objective, a motorized multi-tile scanning strategy was implemented. As illustrated in Fig. S4a, a 5×5 array of individual tiles was acquired within a spatial range centered around $[-0.55\text{ mm}, 0.35\text{ mm}]$ relative to the original center to cover the same region as ($10\times$) objective lens' FOV. We used a translation step size corresponding to approximately 70% of the single-tile FOV dimension for $20\times$ objective.

To register adjacent tiles, Scale-Invariant Feature Transform (SIFT) descriptors were extracted and matched across overlapping regions (Fig. S4b). In a representative tile pair (highlighted by the red box in Fig. S4a, corresponding to row 1, col 2 and row 1, col 3), a total of 5107 matching feature pairs were detected. Following RANSAC filtering, 2732 robust inliers were identified (with 25 representative inliers displayed in green) while 2375 outliers were rejected.

The final stitched result for the M_{00} channel (Fig. S4c) demonstrates excellent alignment and boundary continuity. Notably, the relative displacement between adjacent tiles exhibited a slight diagonal drift rather than an ideal horizontal translation. This subtle geometric shift stems from a minor misalignment between the camera coordinate system and the motorized stage movement directions. Therefore, a manual maximum inscribed rectangle cropping step was applied to remove black boundary regions aiming for best visual effect.

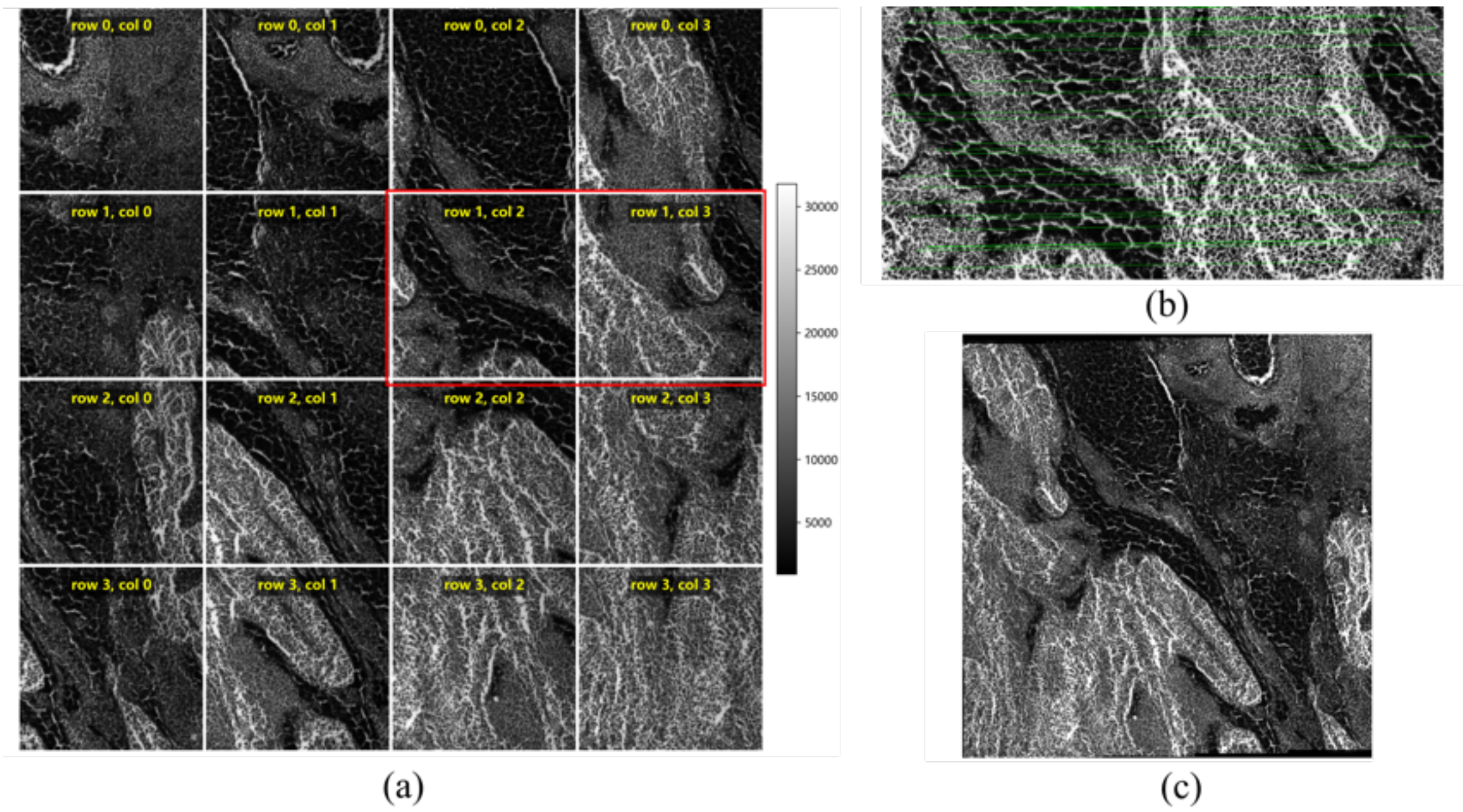

Figure S4. Large field-of-view image stitching pipeline using $20\times$ objective scanning, example results using sample I-22-176-8(A) (**a**) Spatial grid of raw image tiles displaying intensity distribution across a single channel, sharing a common intensity colorbar. To approximate the field of view (FOV) of a $10\times$ objective while retaining high spatial resolution, a 5×5 grid scan was executed in a $0.9mm \times 0.9mm$ region with a step size corresponding to approximately 70% of the single tile length. (**b**) SIFT feature matching between two adjacent tiles (highlighted by the red box in (a): row 1, col 2 and row 1, col 3). Matched feature pairs are connected via green lines. (**c**) Final stitched result for the M_{00} channel after alignment, showing high visual continuity.

2.4 Feature Mask Extraction: Stain-Decoupling and Feature-Composite SLIC

Although mask extraction is not a primary methodological focus of this work, it is required to identify structurally meaningful tissue regions and, importantly, to enable a direct comparison between conventional H&E staining and the proposed pseudo-color (virtual-staining) representation. To account for the distinct characteristics of the two imaging modalities, modality-specific segmentation strategies were employed to generate corresponding masks.

To provide methodological transparency without diverting from the primary contributions of this work, representative examples of both mask-extraction pipelines are presented in the following paragraph. These examples illustrate the processing steps from the original image or multichannel feature matrix to the final

mask and document the practical implementation of the mask extraction and cross-modality comparison procedures.

2.4.1 Stain-Decoupling for H&E image

For H&E histological images, stain separation based on the Macenko method in optical-density space was used to decouple the principal stain components and derive a tissue/stain mask. This H&E-derived mask was used as the reference mask for subsequent assessment of structural correspondence.

Taking the untreated sample I-22-249-4(B) (trt-) as an illustrative example, an unsupervised Macenko stain separation was applied in Optical Density space to decouple the multi-channel input into Hematoxylin (H) and Eosin (E) concentration maps (Fig. S5a–b). Subsequently, Otsu thresholding on the H channel generated a clean binary ROI mask for background suppression (Fig. S5c). In melanoma histopathology, optical density-based stain separation (e.g., Macenko algorithm) plays a crucial role in decoupling Hematoxylin from dark endogenous melanin pigments and background artifacts. The isolated H-channel matrix enhances nuclear contrast, facilitating accurate boundary extraction (ROI masking) and automated quantitative assessment of key diagnostic hallmarks, such as nuclear pleomorphism, mitotic activity, and Breslow depth delineation.

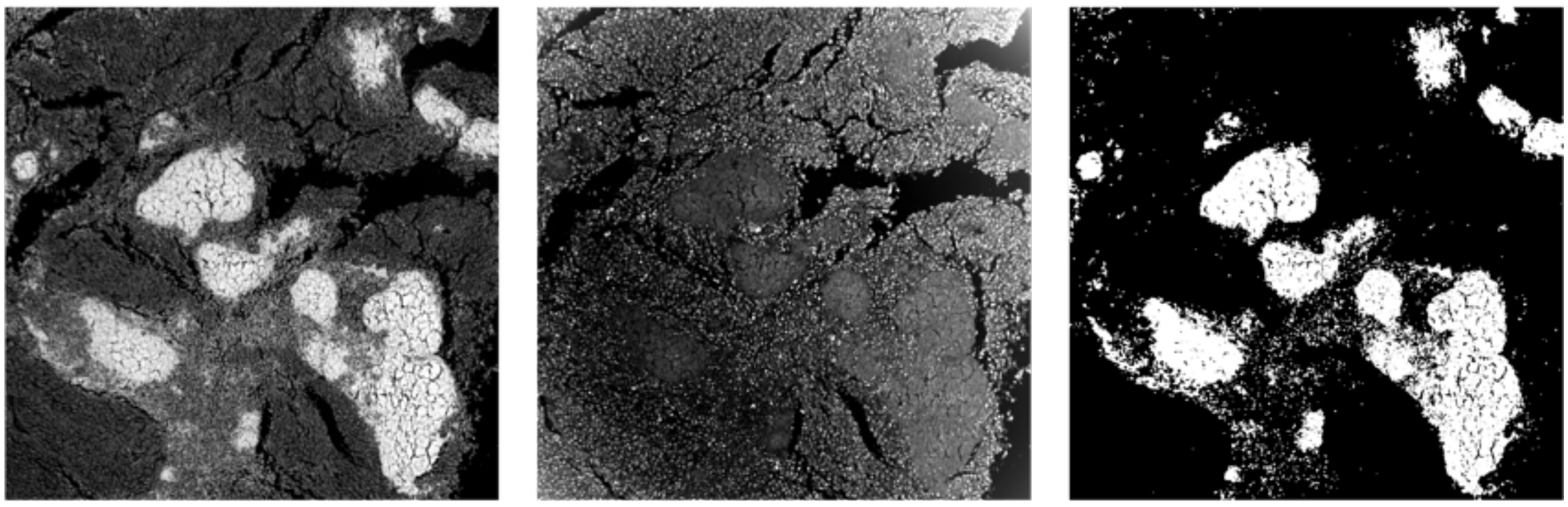

(a) (b) (c)

Figure S5. Stain deconvolution and tissue mask generation from H&E histological imaging. Stain separation based on the Macenko method in optical-density space was applied to decouple the RGB H&E image into its principal chromophore components: (**a**) extracted Hematoxylin concentration map highlighting cell nuclei and dark structures, (**b**) extracted Eosin concentration map corresponding to blood vessels, extracellular matrix and cytoplasm, and (**c**) derived binary tissue/stain mask used as the reference mask for subsequent evaluation of structural correspondence.

2.4.2 Feature-Composite SLIC for pseudo-coloring

For the multichannel polarimetric feature matrices, the corresponding feature channels were first mapped to a pseudo-color representation, followed by Simple Linear Iterative Clustering (SLIC) incorporating both spatial proximity and feature similarity to delineate structurally distinct tissue regions. The resulting segmentation was used to generate the corresponding feature-derived structural mask from the virtual-staining representation.

To facilitate a direct comparative analysis for the reader, Fig S6 presents the polarimetric pseudo-color visualization results, which were cropped from the full stitched map after precise registration with the corresponding 10× single field-of-view (FOV) sub-region. Building upon these mapped channels, Figure S7 presents a visual comparison between the baseline ROI derived from H&E stain decoupling and the structural feature regions extracted from various polarimetric pseudo-color representations using SLIC clustering for the untreated (Trt−) specimen. In Figure S7a, Macenko stain decoupling isolates the primary histological components to establish a reference tissue ROI, capturing the macro-scale spatial boundary of the specimen. Figures S7b–h show the corresponding pseudo-colored polarimetric images alongside their SLIC-segmented feature masks across intra-mechanism (b–d), Lu–Chipman (e), and inter-mechanism (f–h) mapping strategies.

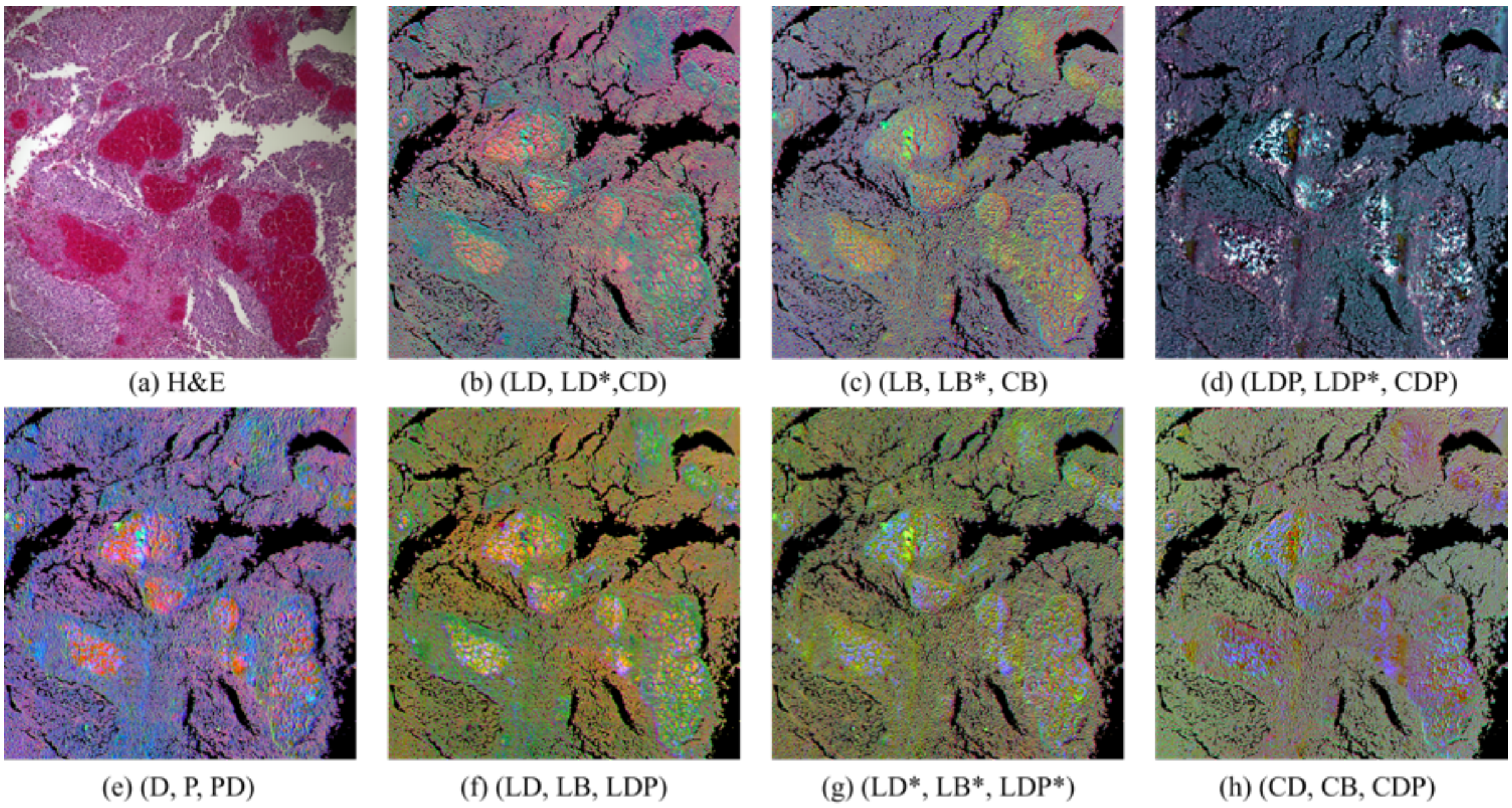

Figure S6. Pseudo-color visualization of polarimetric parameters for the untreated (Trt−) specimen within an extracted region of interest (ROI). The ROI was identified and extracted by precise registration between a large-scale stitched optical map and a corresponding 10× single field of view (FOV). (a) Reference H&E-stained image of the corresponding tumor region within the registered ROI. (b–d) Intra-mechanism mapping, assigning the linear, orientation-resolved linear, and circular components of a single physical mechanism to the R, G, and B channels, respectively: (b) dichroism (LD, LD^*, CD); (c) birefringence (LB, LB^*, CB); (d) depolarization (LDP, LDP^*, CDP). (e) Lu–Chipman decomposition, with overall diattenuation (D), retardance (R), and depolarization (PD) mapped to the R, G, and B channels. (f–h) Inter-mechanism mapping, assigning one representative component from each of the three mechanisms—dichroism, birefringence, and depolarization—to the R, G, and B channels, grouped by component type: (f) linear components (LD, LB, LDP); (g) orientation-resolved linear components (LD^*, LB^*, LDP^*); (h) circular components (CD, CB, CDP).

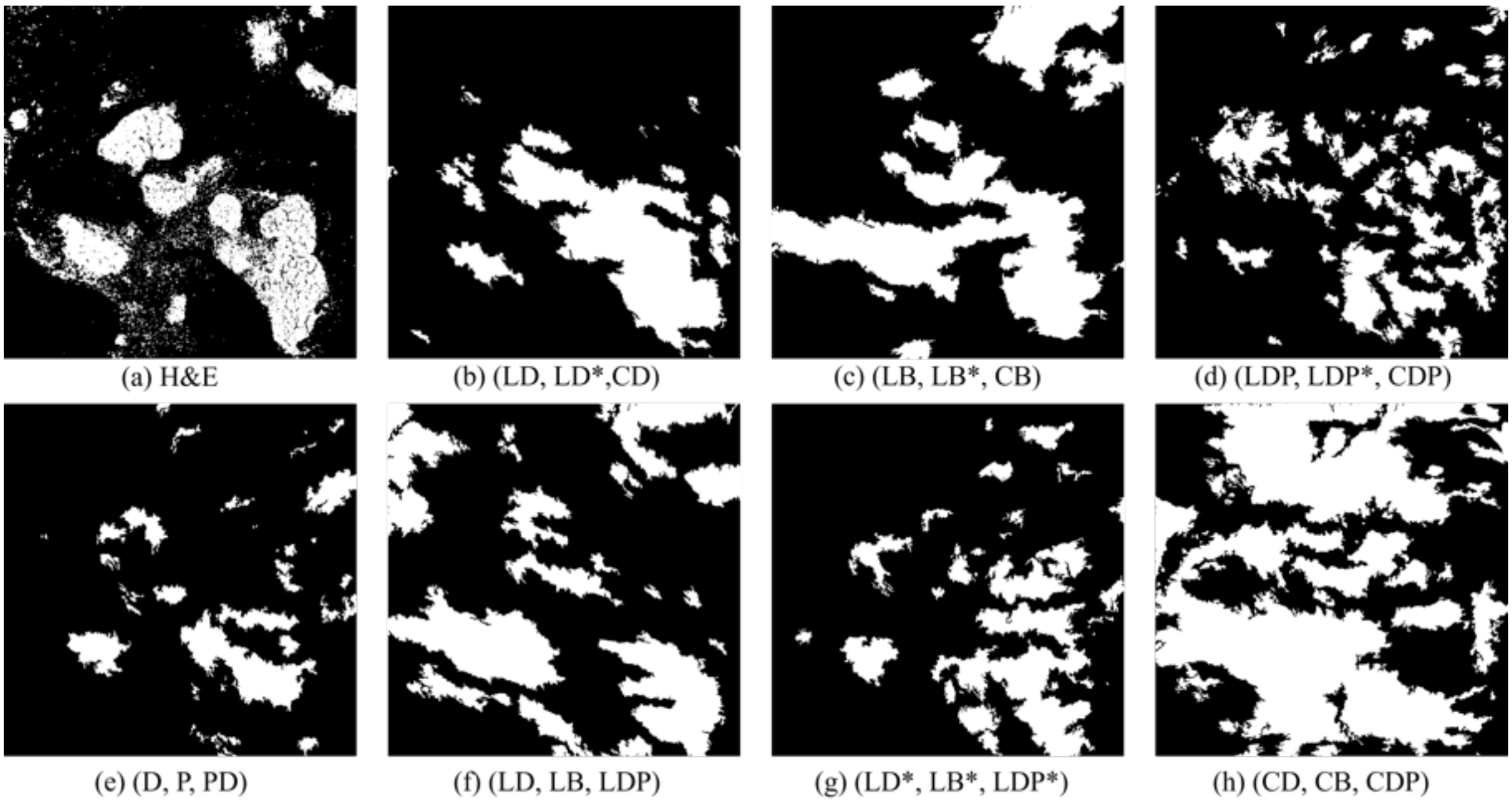

Figure S7. Extracted polarimetric feature regions corresponding to the identical ROI in Fig S6 for the untreated (Trt−) specimen. Panel labels (a–h) follow the same channel mapping configurations as in Fig S6: (a) reference H&E-stained image of the tumor region; (b–d) intra-mechanism mapping for (b) dichroism (LD, LD*, CD), (c) birefringence (LB, LB*, CB), and (d) depolarization (LDP, LDP*, CDP); (e) Lu–Chipman decomposition (D, R, PD); (f–h) inter-mechanism mapping grouped by component type for (f) linear components (LD, LB, LDP), (g) orientation-resolved linear components (LD*, LB*, LDP*), and (h) circular components (CD, CB, CDP).

2.4.3 Dice similarity coefficient computation

The two independently derived masks were spatially registered and compared to quantitatively assess the structural consistency between the H&E and virtual-staining representations. The Dice similarity coefficient (DSC), as defined in the main text, was used to quantify the spatial agreement between the two masks. This analysis provides a direct validation of whether the structural information revealed by the proposed pseudo-color representation is consistent with that observed in conventional H&E staining.

The quantitative indicators for different sample groups have been provided in the main text. Qualitatively, the SLIC-derived masks effectively delineate the salient structural boundaries across most polarimetric representations without requiring explicit histological supervision. Notably, intra-channel dichroism (b) and birefringence (c) as well as orientation-resolved linear components (g) exhibit pronounced spatial fidelity to the H&E-derived reference mask, successfully preserving the underlying tissue architecture. In contrast, representations dominated by depolarization (d) or circular components (h) tend to produce more fragmented or diffuse feature regions, reflecting their sensitivity to localized micro-structural scattering rather than macro-scale histological boundaries. This visual comparison confirms that specific polarimetric pseudo-color schemes can reliably capture key tissue organization while simultaneously highlighting unique physical contrasts complementary to conventional H&E histological staining.

REFERENCES

Aung, P. P., Nagarajan, P., and Prieto, V. G. (2017). Regression in primary cutaneous melanoma: etiopathogenesis and clinical significance. *Laboratory investigation* 97, 657–668

Chan, J. K. (2014). The wonderful colors of the hematoxylin–eosin stain in diagnostic surgical pathology. *International journal of surgical pathology* 22, 12–32

Gil, J. J. and Bernabeu, E. (1985). A depolarization criterion in mueller matrices. *Optica Acta: International Journal of Optics* 32, 259–261

Gil, J. J. and Bernabeu, E. (1986). Depolarization and polarization indices of an optical system. *Optica Acta: International Journal of Optics* 33, 185–189

Hernandez-Aceves, J. A., Cervantes-Torres, J., Torres-Garcia, D., Zuniga-Flores, F. J., Patino-Chavez, O. J., Peña Agudelo, J. A., et al. (2023). Gk-1 effectively reduces angiogenesis and prevents t cell exhaustion in a breast cancer murine experimental model. *Cancer Immunology, Immunotherapy* 72, 3825–3838

Smith, M. H. (2002). Optimization of a dual-rotating-retarder mueller matrix polarimeter. *Applied optics* 41, 2488–2493

Stein, J. E., Soni, A., Danilova, L., Cottrell, T. R., Gajewski, T., Hodi, F., et al. (2019). Major pathologic response on biopsy (mprbx) in patients with advanced melanoma treated with anti-pd-1: evidence for an early, on-therapy biomarker of response. *Annals of Oncology* 30, 589–596

Vivanco-Rojas, O., García-Bermúdez, M. Y., Iturriaga-Goyon, E., Rebollo, W., Buentello-Volante, B., Magaña-Guerrero, F. S., et al. (2020). Corneal neovascularization is inhibited with nucleolin-binding aptamer, as1411. *Experimental Eye Research* 193, 107977
